# The structure and diversity of microbial communities in *Paederus fuscipes* (Coleoptera: Staphylinidae): from ecological paradigm to pathobiome

**DOI:** 10.1186/s40168-022-01456-z

**Published:** 2023-01-20

**Authors:** Bahar Chamankar, Naseh Maleki-Ravasan, Mohsen Karami, Esmaeil Forouzan, Fateh Karimian, Sabah Naeimi, Nayyereh Choobdar

**Affiliations:** 1 Department of Parasitology, Pasteur Institute of Iran, Tehran, Iran; 2 Departments of Zoology Biosystematics, Payame Noor University, East Tehran Centre, Tehran, Iran; 3 Infectious Diseases and Tropical Medicine Research Center, Health Research Institute, Babol University of Medical Sciences, Babol, Iran; 4 GeneMan Genomics Ltd, Shiraz, Iran; 5 Department of Medical Entomology and Vector Control, School of Public Health, Tehran University of Medical Sciences, Tehran, Iran

**Keywords:** *Pseudomonas*-like *Paederus fuscipes* endosymbiont, Gut microbiota, Genital microbiota, Dermatitis linearis, Pederin, *Wolbachia*, *Spiroplasma*, *Apibacter*

## Abstract

**Background:**

*Paederus fuscipes* is medically the most famous rove beetle, which causes dermatitis or conjunctivitis in humans, as well as gastrointestinal toxicosis in livestock, via releasing toxic hemolymph containing pederin. Pedrin biosynthesis genes have been identified in uncultured *Pseudomonas*-like endosymbionts that are speculated to be acquired through a horizontal transfer. However, the composition of the *P. fuscipes* microbial community, especially of the gut and genital microbiome, remains unclear. This study was aimed to characterize the structure and diversity of *P. fuscipes*-associated bacterial communities in terms of gender, organ, and location using the Illumina HiSeq platform in the southern littorals of Caspian Sea.

**Results:**

The OTUs identified from *P. fuscipes* specimens were collapsed into 40 phyla, 112 classes, 249 orders, 365 families, 576 genera, and 106 species. The most abundant families were Pseudomonadaceae, Spiroplasmataceae, Weeksellaceae, Enterococcaceae, and Rhizobiaceae, respectively. Thirty top genera made up > 94% of the *P. fuscipes* microbiome, with predominating *Pseudomonas*, followed by the *Spiroplasma*, *Apibacter*, *Enterococcus*, *Dysgonomonas*, *Sebaldella*, *Ruminococcus*, and *Wolbachia*. Interesting dissimilarities were also discovered within and between the beetle microbiomes in terms of genders and organs. Analyses showed that *Spiroplasma* / *Apibacter* as well as *Pseudomonas* / *Pseudomonas* were the most abundant in the genitals / intestines of male and female beetles, respectively. Bacterial richness did not display any significant difference in the three provinces but was higher in male beetles than in females and more in the genitals than intestines.

**Conclusions:**

The present study identified *Pseudomonas*-like endobacterium as a common symbiont of *P. fuscipes* beetles; this bacterium begins its journey from gut and genitalia of females to reach the male rove beetles. Additionally, male and female rove beetles were characterized by distinctive microbiota in different organs, likely reflecting different functions and/or adaptation processes. Evidence of the extension of *P. fuscipes* microbiome from the environmental paradigm to the pathobiome was also presented herein. A comprehensive survey of *P. fuscipes* microbiome components may eventually lead to ecological insights into the production and utilization of defensive compound of pederin and also the management of linear dermatitis with the use of available antibiotics against bacterial pathogens released by the beetles.

Video Abstract

**Supplementary Information:**

The online version contains supplementary material available at 10.1186/s40168-022-01456-z.

## Introduction

Insects are the most abundant and diverse class of animals and one of the most successful groups of living organisms that occupy a variety of ecosystems on earth [1–4]. The key factor in the success of insects is undoubtedly their close relationships with microorganisms, especially bacteria, which frequently affect their adaptability. While insects can easily acquire varied bacteria as they feed, move, and spread in the ecosystems, the rest of the microorganisms is inherited vertically from parents [5–7]. The obtained microbes are not certainly pathogenic but are often beneficial or even essential for the host. Hence, most of the cells of a healthy insect are microbial, the reason why insects are generally considered as multiorganismal animals [8].

Symbiotic bacteria can provide a surprising set of advantages to the host insects. These benefits varies from nutrient provisioning, protection against pathogens, and natural enemies to participation in intra- and inter-specific host communications, impact on pathogen transmission efficiency, management of mating behavior and reproductive strategies, coping with environmental stress, and even pesticide detoxification [8–13]. Despite the fact that only less than 2% of bacteria can be cultivated [14, 15], there is limited information on remaining intracellular and uncultured bacteria that many insect species are dependent on for their survival and reproduction [16, 17].

In the host population, numerous symbionts occur in the form of one or two endosymbionts [18]. Symbionts are generally subdivided as primary or secondary, considering whether they are essential for host survival or improve the extra demands of the host [8]. Unlike the primary symbionts that normally reside in specialized cells, the secondary symbionts do not normally exist in particular organs and can be detected in primary bacteriocytes [19], secondary bacteriocytes and sheath cells [20], salivary glands [21], hemolymph [22], Malpighian tubules [23], and genitals [24]. Among all groups of insects, the secondary symbionts have much less been studied than the primary ones, among which *Wolbachia*, *Spiroplasma*, *Cardinium*, and *Arsenophonus* are dominant bacteria, and about one-third of the arthropod species surveyed harbor at least one of these four species [25].

Rove beetles (Coleoptera: Staphylinidae) are a species-rich group of the suborder Polyphaga with more than 63,000 known species worldwide [26]. At present, the large genus *Paederus* Fabricius, 1775, covers ~ 490 species globally [27], with 14 species in Iran. Among these species, *P. balachowskyi*, *P. balcanicus*, *P. duplex*, *P. fuscipes*, *P. littoralis*, and *P. riparius* have been detected in the Southern Caspian Sea Provinces, i.e., Guilan, Mazandaran, and Golestan [28]. The most notable species of the *Paederus* in the old world is *P. fuscipes* Curtis, 1840, which is distributed from the eastern islands of Britain throughout the Central Asia to Japan and from the southeast to Australia [29]. This species has been reported in Iran from 10 provinces, including three provinces in the Southern littorals of the Caspian Sea [28].

Ecologically, rove beetles play a pivotal role in the natural ecosystems as they spread across a wide range of habitats and interrelate with the diverse trophic levels of the food chain, from producers to consumers and decomposers. In moist ecosystems, these beetles are associated with numerous arthropods, plants, fungi, decomposing litters, mollusks, and vertebrates. Most of them act as the predators of arthropods, and some species have a link to social insects. However, others are scavengers on decaying plant matter or coexist in the nests of rodents [30].

Medically, *Paederus* spp. are the most famed beetles causing significant human injuries [27]. The beetles do not bite or sting but do release their toxic hemolymph containing pederin (C_25_H_45_NO_9_; MM: 503.63; LD_50_: 0.14 mg/kg rat i.p.) when crushing on human skin, thereby inducing dermatitis or conjunctivitis with mild to severe symptoms [31, 32]. The contact dermatitis is a distinct form of stimulus that can be detected with the rapid onset of erythematous lesions. The erythemas may last for a month or more and accompanied by other symptoms such as fever, edema, neuralgia, arthralgia, and vomiting [33, 34]. Pederin is acknowledged as an agent possessing antitumor and antiviral properties [35], presumably through the prevention of DNA replication and protein synthesis [31]. The toxin is originally used by rove beetles as a defensive compound against predators [36].

*P. fuscipes* dermatitis has been reported from China, India, Indonesia, Iran, Italy, Japan, Laos, Russia, Taiwan, Thailand, and Vietnam [29]. Natives in the southern provinces of the Caspian Sea are quite familiar with *Paederus* and also with dermatitis caused so that the beetles are called locally as “Dracula,” “Band,” and “Tan-Gizi” in Guilan, Mazandaran, and Golestan Provinces, respectively. From March to October 2002, a number of 100 patients with *Paederus* dermatitis were referred to Sari dermatological clinics [37]. A total of 156 patients with dermatological lesions were identified in Guilan Province from May to October 2001. The peak of the disease was reported in September, and the face and neck were the most organs involved [38]. There were 94 *Paederus* dermatitis patients referred to clinics and hospitals in Behshahr, from the beginning of September to the end of October, 2000 [39]. There are no official reports on the annual rate of *Paederus* dermatitis in Iran, but based on the available evidence, it can be speculated that the number of cases is very high in the region. Even livestock such as horses and cattle are not immune to the *Paederus* species. They may be poisoned following the accidental ingestion of *P. fuscipes* with forage; therefore, pederin can cause serious damages to the gastrointestinal tract mucosa [29].

Pederin and its analogs (pseudopederin and pederone) have been shown to be synthesized by uncultured *Pseudomonas*-like *P. fuscipes* endosymbiont (*PLPFE*) harbored in the accessory glands of the female genitalia [40, 41]. The bacterium is circulated in the life history of rove beetles, both through the transovarial and horizontal routes [42, 43]. The region containing a genomic island spanning 71.6 kb harboring putative pederin biosynthesis genes (*ped* cluster) has been identified in *P. fuscipes* and indicated that *Pseudomonas*-like endobacterium has acquired the *ped* genes by horizontal transfer path [44].

Despite the great ecological and medical significance, *P. fuscipes* microbiome has not yet fully been studied. Very few surveys have hitherto been performed on *P. fuscipes* microbiota, with focusing mainly on *Wolbachia* and *Pseudomonas*-like endosymbionts [43, 45]. Microbiome studies require high-quality data, such as *16S rRNA* amplicons, to produce valid results. High-performance comparative metagenomic methods, with the development of next-generation sequencing (NGS) platforms, have led to an eruption of research efforts that have accelerated our understanding of the composition and function of bacterial populations in highly diverse environments [46]. With this background, the present study was performed to scrutinize the types of bacteria and their distribution in *P. fuscipes* beetles in terms of genders (male and female), organs (gut, genitalia, and the total body), and locations (Guilan, Mazandaran, and Golestan). In general, the hypotheses examined in this study were what kind of bacteria does *P. fuscipes* have and what are their circulation path in the beetle population? To this end, large-scale data were generated from *P. fuscipes* microbiome, highlighting the role of bacteria in the beetle’s life history and medical importance.

## Methods

### Specimen collection and processing procedure

Live adult rove beetles were gathered in June–July 2019–2020 using hand catch method in wet areas, especially from the paddy fields of three northern provinces of Iran, including Guilan, Mazandaran, and Golestan, where the highest cases of linear dermatitis caused by *Paederus* spp. have been reported [37–39]. Captured beetles were classified into two groups: (1) the group whose whole body was the subject of the study and therefore immersed in ethanol immediately after collection and (2) the group whose gastrointestinal and reproductive organs had to be dissected; thus, the insects were taken alive to the laboratory (Additional file 1: Figure S1). Species and gender of both groups were determined using available identification keys [47–49] and photography. The aedeagus of representative male specimens was dissected and mounted on slides for further species verifying (Additional file 1: Figure S2).

Following taxonomic identification, for the specimens of the first group, possible microbes and foreign particles adhering to the exocuticle were removed three times with the aid of 70% ethanol and centrifugation and then were kept in ethanol at −20 °C. For the specimens of the second group, male and female beetles were first immobilized by keeping at −20 °C for 5 min. Thereafter, each beetle was thoroughly washed three times by spraying 70% ethanol with high pressure. The specimens were eventually microdissected within normal saline on a microscopic slide under aseptic conditions. The whole parts of the intestine (including foregut, midgut, and hindgut) and genitalia (including gonads and glands) of male and female beetles were cut up and deposited in 70% ethanol at −20 °C until DNA extraction (Additional file 1: Figure S1).

### DNA extraction, PCR, and amplicon sequencing

The specimens/tissues were precipitated by a high-speed centrifugation, and their alcohol was entirely removed. DNA extraction from alcohol-free samples was performed using QIAamp DNA Micro Kit (ID: 56304, Qiagen, Germany) according to the protocol of genomic DNA extraction from tissues. RNA contamination in the specimens was eliminated by adding 2 μl of RNase A (10 mg/ml; Fermentas, USA) to each 20 μl of DNA dissolved in Tris-EDTA buffer (pH 8.0), as well as by incubating at 37 °C for 3–4 h. DNAs of specimens were mixed by sex and also by the field sampling location of the beetles from each province.

The DNA of a male as well as a female beetle was selected from each location for microbiome analysis. For each province, a specimen of the pooled DNA of male (OD 153.634–683.326 ng/μL) and female (OD 248.578–657.784 ng/μL) beetles were prepared for sequencing. The DNA of digestive and reproductive tissues of 38 male and 38 female beetles from the Amol city, Mazandaran Province, was examined. The DNA of two beetles, including one male (from Amol) and one female (from Khalil-Shahr), was likewise investigated individually (Additional file 1: Table S1). Overall, the DNA of 84 beetle specimens was selected for microbiome analysis.

A 466-bp portion of the *16S rRNA* gene was amplified using the primers 341F (5′-CCTAYGGGRBGCASCAG-3′) and 806R (5′-GGACTACNNGGGTATCTAAT-3′) covering the V3–V4 hypervariable regions of bacteria and archaea [50]. Quadruplicate 25-μl reactions were run for 35 cycles of amplification involving 5 s at 98 °C, 20 s at 56 °C, and 20 s at 72 °C using Titanium Taq DNA Polymerase (Clontech, Takara, Japan). The pooled amplicons were purified using QIAquick PCR Purification Kit (Qiagen, Germany). The high-quality NGS were accomplished by Beijing Genomics Institute (BGI) in China, with error rates of less than 1%. The metagenomic libraries were prepared in accordance with the previously reported protocols [51].

### Bioinformatic processing and statistical analysis

The raw sequences was analyzed as per the standard guidelines of paired end-data with QIIME2 v. 2018.11 [52] pipeline. Initially, the quality of raw sequences was checked by FastQC program v. 0.11.9 [53]. The sequences of primers and adapters along with low-quality sequences were removed from the reads using cutadapt v. 2018.11.0 [54] and Trimmomatic v. 0.40 [55], respectively. Then the fixed sequenced paired end reads were integrated to make larger amplicons (~ 420 bp) by applying PEAR tool [56]. Amplicons with sequence similarity ≥ 97% were selected as input to create the taxonomic annotation and to form the operational taxonomic units (OTUs) table based on the Open-Reference method. The reads were categorized using the Qiime2 feature-classifier classify-sklearn method [57]. Eventually, the SILVA ribosomal RNA gene database (v.123) was utilized for taxonomic assignment of the categorized reads [58]. The microbial communities of each specimen were determined from the mentioned assignments, from phylum to species levels.

To determine the suitable sequencing depth for each specimen, a rarefaction curve was plotted for comparisons. The alpha diversity, which essentially determines the number and distribution of bacteria in each specimen, was calculated using four indices, comprising of Shannon’s entropy [59], observed features, Faith’s phylogenetic diversity (PD) [60], and Pielou’s evenness [61] in the Qiime2 package. Analysis of dissimilarity between specimens, which in fact is the measure of population composition, i.e., beta diversity, was achieved using Bray-Curtis [62], Jaccard [63], unweighted UniFrac, and weighted UniFrac distances [64] at a sub-sampling depth of 100 sequences per sample. The α / β-group significances were evaluated by the non-parametric Kruskal–Wallis and permutation multivariate analysis of variance (PERMANOVA) analyses, respectively.

The relative abundance of bacteria across samples was compared with STAMP (Statistical Analysis of Metagenomic Profiles) bioinformatics software v. 2.1.3 [65], using the OTU table from Qiime pipeline without any rarefaction. Parameters of statistical analysis were adjusted to fit any type of comparisons of the divergences among multiple groups, two groups and two samples. Statistical significance of the differences between multiple groups were calculated using ANOVA test and Games-Howell post hoc test at 0.95 confidence interval, and corresponding *p* values were corrected by Storey’s FDR approach. To compare dissimilarities between two groups / two samples, the parameters were respectively set as Welch’s *t*-test / Fisher’s exact test and DP: Welch’s / DP: Newcombe-Wilson confidence inverted methods with the same confidence interval, *p* value, and correction methods mentioned above. Unclassified sequences were deleted during analysis with STAMP software.

The composition of bacteria was visualized by Circos tool (v. 0.69-6) based on the elements of the beetle gender, body parts, and sampling locations, to facilitate the identification and analysis of similarities and differences arising from sequence comparisons [66]. Online Cytoscape software [67] was also used to further classify and quantify the shared and exclusive bacteria in the gut and genitalia of male and female beetles. Additionally, Venn diagrams [68] were also generated to observe the partition of the bacteria genera across the *P. fuscipes* body parts / locations sampled.

## Results

### Large-scale sampling of *P. fuscipes*

Staphylinid materials were collected from 23 locations of three provinces of southern coast of Caspian Sea, from Rezvanshahr to Aliabad Katoul, at a distance of ~ 600 km. In total, 464 (243 male and 221 female) *P. fuscipes* beetles were captured from Guilan (*n* = 169), Mazandaran (*n* = 240), and Golestan (*n* = 55) Provinces (Additional file 2: Table S1).

### Taxonomic profiling of *P. fuscipes* metagenomic sequences

Illumina HiSeq paired end sequencing generated a total of 1,030,496 bacterial *16S rRNA* gene sequences from all 12 *P. fuscipes* materials after trimming and quality control (Additional file 1: Tables S2-S5). The mean read depth was 85,875 per specimen with a minimum of 27,313 and a maximum of 113,994 for the female and the male genitalia, respectively (Additional file 1: Tables S2 and S6). Rarefaction analysis showed that all curves reached the plateau phase, which indicates the full sampling of the microbial communities (Additional file 1: Figure S3).

**Fig. 1:**
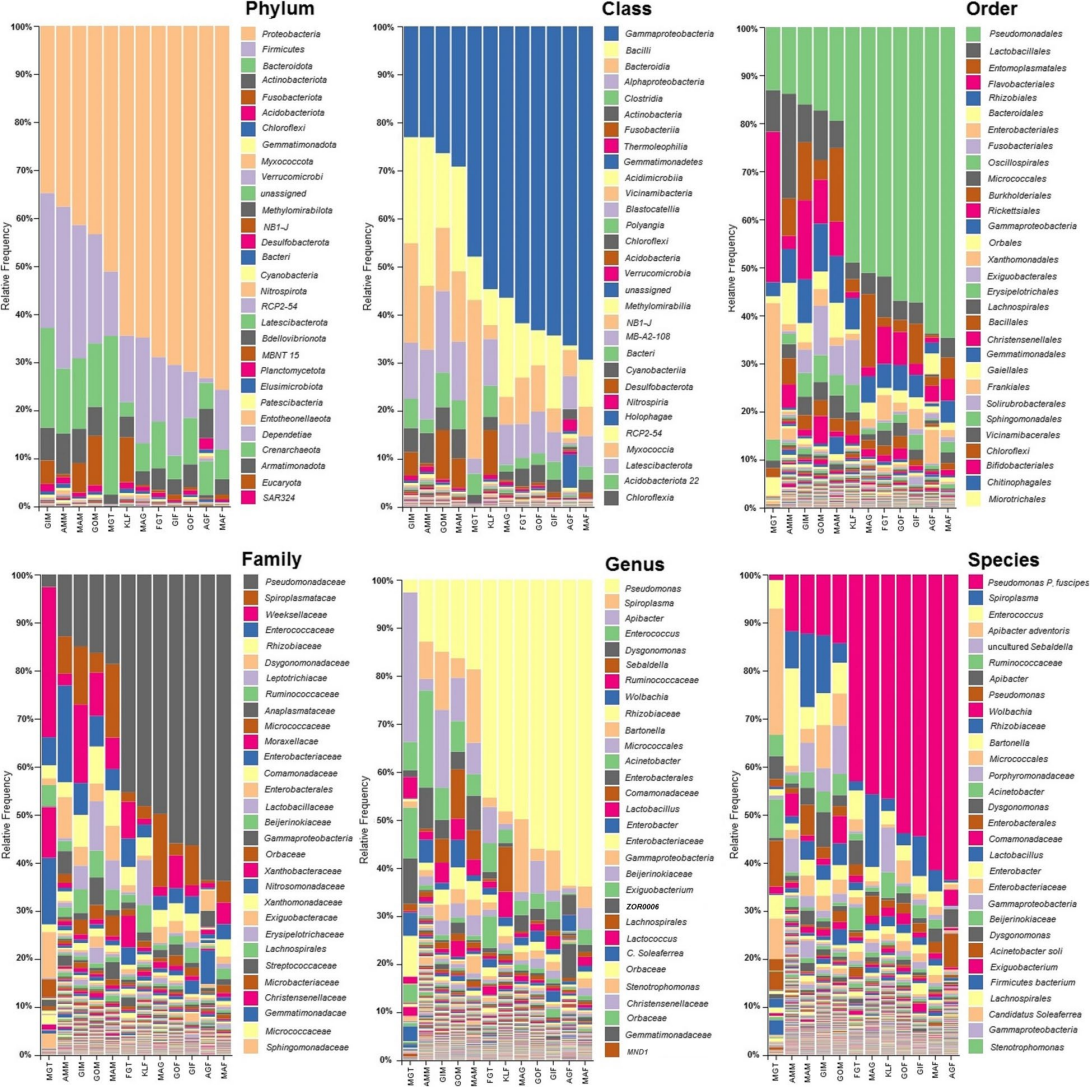
Relative frequency and taxonomical composition of microbiome of *Paederus fuscipes* beetles by phylum, class, order, family, genus, and species. Legends are shown for 30 OTUs with high frequency

Overall, the OTUs identified from *P. fuscipes* specimens belonged to 40 phyla, 112 classes, 249 orders, 365 families, 576 genera, and 106 species (Additional file 1: Table S6). The most abundant phyla were Proteobacteria (x̄ ~ 58%; 38–76%), Firmicutes (x̄ ~ 18%; 1–34%), Bacteroidota (x̄ ~ 12%; 3–33%), Actinobacteriota (x̄ = 5%; 2–8%), and Fusobacteriota (x̄ ~ 3%; 0–12%), with the main bacteria classes Gammaproteobacteria (x̄ ~ 48%; 23–69%), Bacilli (x̄ ~ 14%; 1–31%), Bacteroidia (x̄ ~ 12%; 3–33%), Alphaproteobacteria (x̄ ~ 9%; 3–17%), and Clostridia (x̄ ~ 4%; 0.05–7%), respectively (Fig. 1, Additional file 1: Table S7). The most plentiful families were Pseudomonadaceae (x̄ ~ 33%; 3–64%), Spiroplasmataceae (x̄ ~ 12%; 0.06–15%), Weeksellaceae (x̄ ~ 8%; 1–31%), Enterococcaceae (x̄ ~ 5%; 0.29–20%), and Rhizobiaceae (x̄ ~ 4%; 0.19–8%). At the genus level, the top 30 genera accounted for more than 94%; among them, *Pseudomonas* was dominated, which constituted nearly 37%. Moreover, seven genera, *Wolbachia*, *Ruminococcus*, *Sebaldella*, *Dysgonomonas*, *Enterococcus*, *Apibacter*, and *Spiroplasma*, individually accounted for 2–7%, and the remaining genera each constituted <2% (Fig. 1, Additional file 1: Table S7). At the species level, the top five species accounted for more than 99%. Among these species, *PLPFE* had the highest abundance, and *Apibacter adventoris* was relatively rich, which constitute about 35 and 5%, respectively. The *Acinetobacter soli* and a Firmicutes bacterium accounted for 0.57% and 0.37%, respectively (Fig. 1; Table S7).

### Diversity of the bacterial communities in *P. fuscipes*

**Fig. 2:**
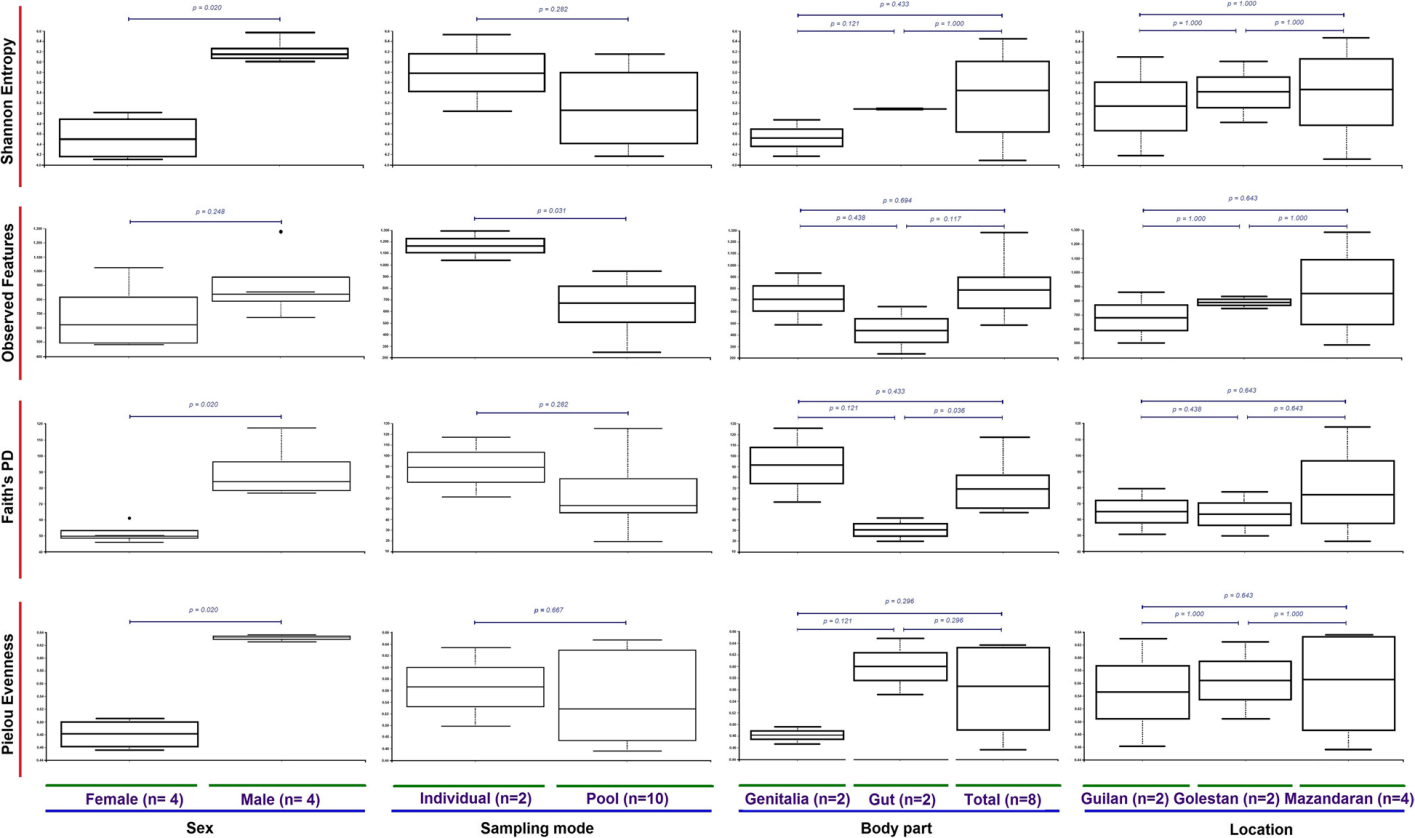
Box plots of alpha diversity indices (Shannon’s entropy, observed features, Faith’s PD, and Pielou’s evenness) comparing microbiome in terms of gender, sampling modes, body parts, and locality of studied *Paederus fuscipes* specimens

The overall results of the research showed differences in the structure of microbial communities within and between the specimens. Comparisons of alpha diversity indices using Kruskal–Wallis statistic demonstrated significant variations between the studied microbiomes. In terms of gender, three indices of Shannon’s entropy (*p* = 0.020), Faith’s PD (*p* = 0.020), and Pielou’s evenness (*p* = 0.020), in terms of sampling mode and body parts (i.e., gut versus the total body), the indices of observed features (*p* = 0.031) and Faith’s PD (*p* = 0.036), respectively, showed significant divergences between analyzing components (Fig. 2).

Shannon’s diversity, which measures species richness (numbers of distinct taxa) and evenness (similarity of abundance), averaged 6.18 for males and 4.50 for females. The index for individual and collective sampling modes was calculated as 5.77 and 5.05, and for the total bodies, guts, and genitalia were measured as 5.34, 5.12, and 4.52, respectively. This quantity also estimated to be 5.44, 5.39, and 5.14 for Golestan, Mazandaran, and Guilan Provinces, respectively (Fig. 2).

The mean observed species richness for males and females were determined as 908 and 687 OTUs, while for individual and collective sampling modes, the measures were 1152 and 637 OTUs, respectively. A number of 797 OTUs was identified for the total bodies, 708 for the genitalia and 438 for the guts. Regarding the location field, 865, 784, and 676 OTUs were detected for Mazandaran, Golestan, and Guilan Provinces, respectively (Fig. 2).

Faith’s PD, which integrates phylogenetic relationships to offer an evolutionary measure of biodiversity, calculated 91 OTUs for male and 52 OTUs for female beetles, as well as 89 OTUs for individual and 63 OTUs for collective sampling modes. Regarding different parts of the body, 90 OTUs were identified for the genitalia, 71 for the total bodies, and 30 for the guts. Concerning the location field, 78, 64, and 63 OTUs were determined for Mazandaran, Guilan, and Golestan Provinces, respectively (Fig. 2).

Pielou’s evenness, which measures the similarity of the abundance of different taxa (distinct sequences) within each specimen, averaged 0.63 for males and 0.48 for females. This index was calculated as 0.57 and 0.55 for individual and collective sampling modes, respectively. This quantity was determined to be 0.60, 0.56, and 0.48 for the guts, total bodies, and genitalia, respectively. It was also measured as 0.56, 0.56, and 0.55 for the Mazandaran, Golestan, and Guilan Provinces, respectively (Fig. 2).

Alpha diversity indices showed changes in the gut and genital microbiome of beetles of both sexes (Table 1). The observed species richness and Faith’s PD indices in the stomachs of females were 2.72 and 2.14 times higher than those of males, respectively. In male genitalia, the observed species richness index was 1.93 times that of females, and the Faith’s PD index in female genitalia was 2.37 times that of males. The descriptive microbiome values of the intestinal / genital communities are detailed in the Table 1.

**Table 1:** Alpha diversity indices calculated for the microbiomes of 38 pooled guts and genitals of male and female *Paederus fuscipes*

Indices/compartment	Gut		Genitalia	
	Male (MGT)	Female (FGT)	Male (MAG)	Female (AGF)
Shannon’s entropy	5.10	5.14	4.90	4.15
Observed features	236	641	933	484
Faith’s PD	19.20	41.12	55.92	125.07
Pielou’s evenness	0.65	0.55	0.50	0.47

Comparisons of beta diversity indices using PERMANOVA statistic showed significant variations among the microbiomes of genders and body parts. Bray-Curtis and unweighted/weighted UniFrac dissimilarities suggested that bacterial composition differed between female and male beetles (PERMANOVA; *F* = 5.80, *p* = 0.029 / *F* = 2.21, *p* = 0.027 / *F* = 12.59, *p* = 0.024; Table 2). Jaccard distance exhibited that the bacterial structure of genitalia varied from the total body (PERMANOVA; *F* = 1.15, *p* = 0.044; Table 2). Also, Bray-Curtis and unweighted UniFrac measures displayed that the bacterial communities of guts diverged from total bodies (PERMANOVA; *F* = 2.55, *p* = 0.045 / *F* = 1.57, *p* = 0.049; Table 2). However, the microbiome was similar between other fields of investigated (PERMANOVA; *F* = 2.86, *p* > 0.05; Table 2).

**Table 2:** Pseudo *F* table of PERMANOVA analysis based on Bray-Curtis, Jaccard, and unweighted/weighted UniFrac dissimilarities

Group 1	Group 2	Sample size	Bray-Curtis		Jaccard		Unweighted UniFrac		Weighted UniFrac	
			Pseudo ***F***	***p*** value	Pseudo ***F***	***p*** value	Pseudo ***F***	***p*** value	Pseudo ***F***	***p*** value
Female	Male	8	5.803874	**0.029**	1.022455	0.325	2.219573	**0.027**	12.592005	**0.024**
Individual	Pool	12	0.644945	0.528	1.081579	0.196	0.950704	0.521	0.281726	0.531
Genitalia	Gut	4	1.906539	0.361	1.034930	0.316	1.098701	0.656	1.445207	0.326
Genitalia	Total body	10	2.172484	0.196	1.152574	**0.044**	1.107672	0.375	6.463713	0.105
Gut	Total body	10	2.552593	**0.045**	1.146777	0.101	1.573647	**0.049**	2.463113	0.053
Guilan	Golestan	4	0.529113	0.670	1.062011	0.315	0.876644	0.640	0.281789	0.670
Guilan	Mazandaran	6	0.330159	0.944	0.945451	0.921	0.627110	0.949	0.172155	0.922
Golestan	Mazandaran	6	0.559537	0.821	1.059570	0.136	1.027351	0.380	0.226587	0.935

Significant *p* values (≤0.05) are bolded

**Fig. 3:**
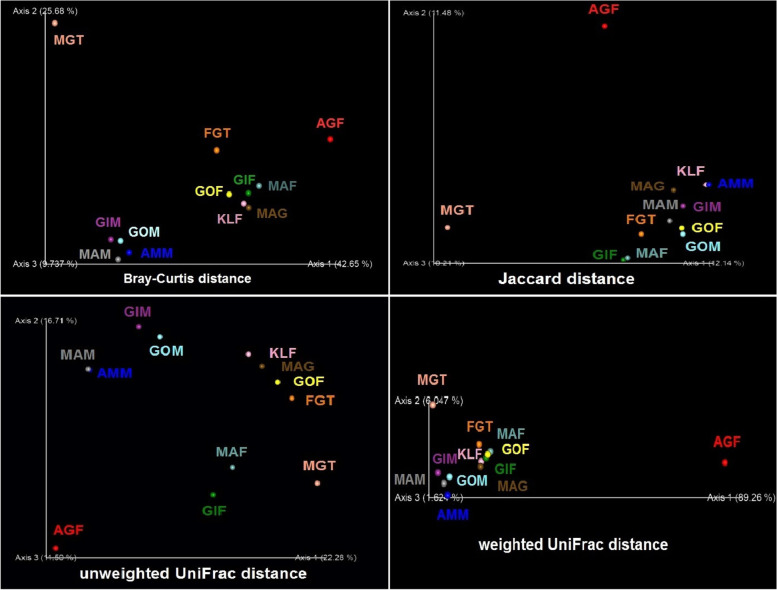
Principal coordinates analysis (PCoA) plots of beta diversity distances (Bray-Curtis, Jaccard, and unweighted/weighted UniFrac) between *Paederus fuscipes* specimens. Distances between points on the ordination plot reflect relative dissimilarities in microbiome structures. Eigenvalues of PCoA1, PCoA2, and PCoA3 are shown in parentheses. Samples were colored according to the fields sources

The inconsistency between different structures of bacterial communities was determined using principal coordinate analysis (PCoA) ordinations (Fig. 3). In general, the PCoA ordinations demonstrated that the microbiomes of male and female beetles are taxonomically quite different owing to their tendency to cluster at certain points on the chart. Also, the microbiomes of the reproductive organs of females as well as digestive tracts of males were divergent from other specimens (Fig. 3). These sharp discrepancies are mostly affected by the abundant presence of *Pseudomonas* and *Apibacter*, respectively. A global representation of bacterial clustering investigated in this study is illustrated in Fig. 3.

### Bacterial communities of gastrointestinal tracts of *P. fuscipes*

A total of 130,134 bacterial sequences were obtained from the gastrointestinal tract of male (*n* = 37,417) and female (*n* = 92,717) *P. fuscipes*. Sequences identified from the gastrointestinal tract of male beetles were classified into 87 families, 103 genera, and 10 species. The identified sequences of the female gastrointestinal tract were also classified into 174 families, 203 genera, and 30 species (Additional file 2: Table S6). Most OTUs in the guts of male and female beetles belonged to the Weeksellaceae and Pseudomonadaceae families, respectively (Additional file 2: Table S7).

**Fig. 4:**
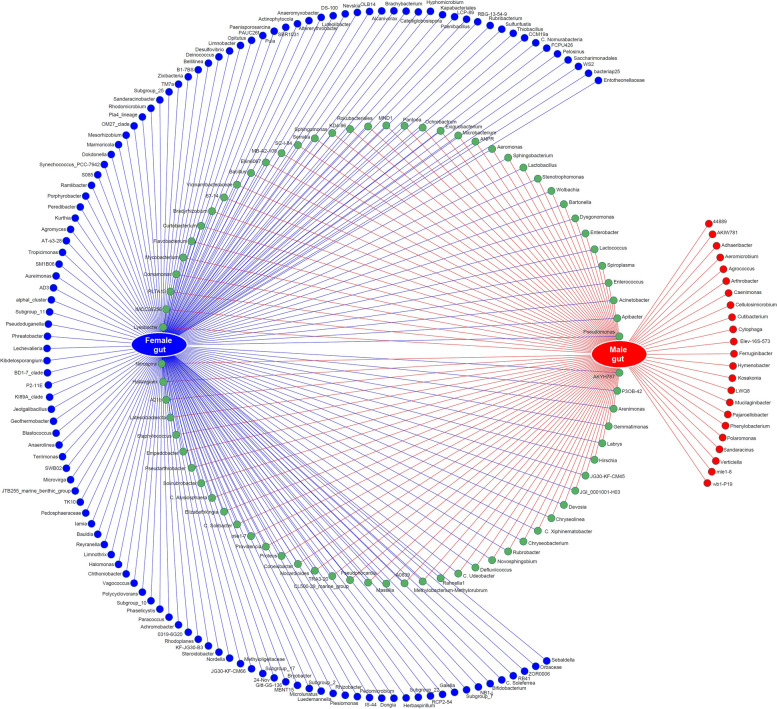
Network analysis showing the shared (green circles crossed by both red/blue lines) and non-shared gut bacteria of male (red circles) and female (blue circles) *Paederus fuscipes* specimens

**Fig. 5:**
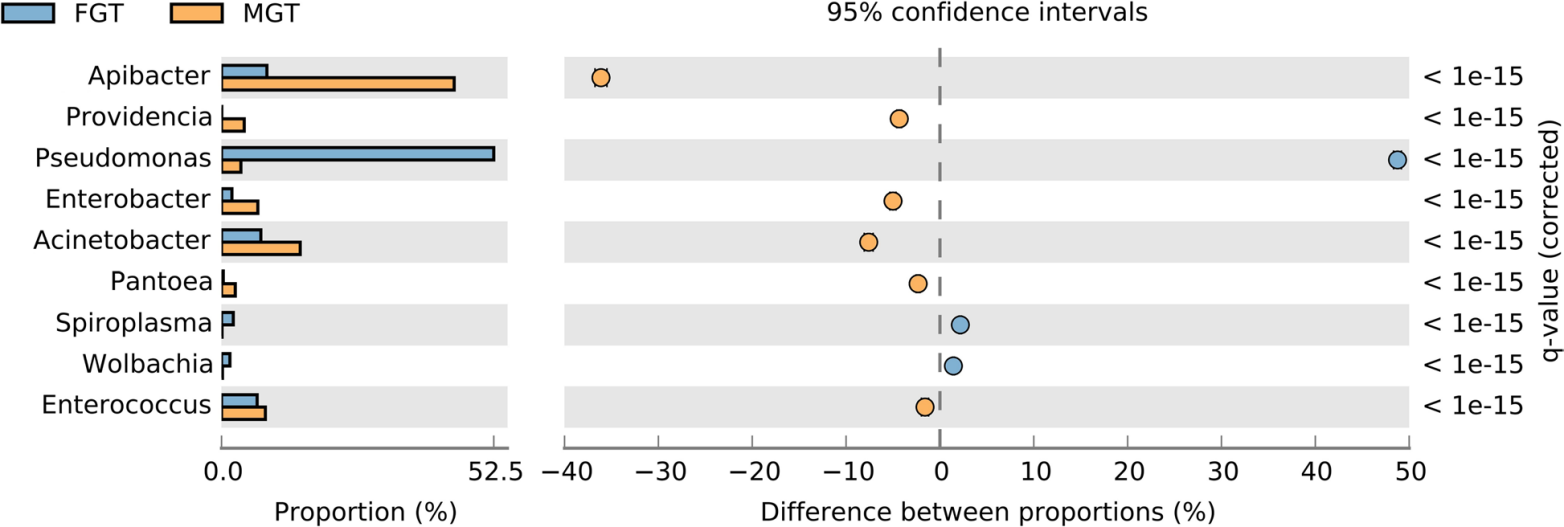
Comparison of gut bacteria profile of male and female *Paederus fuscipes* specimens using STAMP analysis. Comparisons were made at the bacterial genus level. Corrected *p* values were calculated based on Fisher’s exact test method using Storey’s FDR approach. *P* values < 0.05 were taken for comparison. The bar plot indicated in blue or orange shows a positive or negative difference between read proportions. The size effect of >100 reads is included in the comparisons. Differences between samples are shown at 95% confidence intervals

Network analysis of bacteria at the genus level showed that of 221 genera of bacteria known in *P. fuscipes* guts, 121 and 23 were exclusively present in female and male stomachs, respectively, and 77 were shared between both sexes (Fig. 4). STAMP analysis of gastrointestinal tracts of male and female beetles reflected that *Pseudomonas* is present in greater abundance in females and lesser abundance in males with positive differences, whereas *Apibacter* is less abundant in females and more abundant in males with negative differences (Fig. 5). *Wolbachia* / *Spiroplasma* were found in the gastrointestinal tract of male and female beetles with the relative frequency of 0.12 / 0.06% and 1.39 / 1.96%, respectively. At the species level, the most copious bacteria in the guts of male and female insects were *Apibacter adventoris* and *PLPFE*, respectively.

### Bacterial communities of reproductive organs of *P. fuscipes*

The number of bacterial reads of the reproductive organs of male beetles (*n* = 113,994) was about four times that of female insects (*n* = 27,313). The sequences identified from the reproductive tissues of male *P. fuscipes* were classified into 216 families, 278 genera, and 25 species. The identified sequences of the female reproductive tissues were also classified into 138 families, 146 genera, and 9 species (Additional file 2: Table S6). Most OTUs in the genitals of both male and female beetles belonged to the Pseudomonadaceae family.

**Fig. 6:**
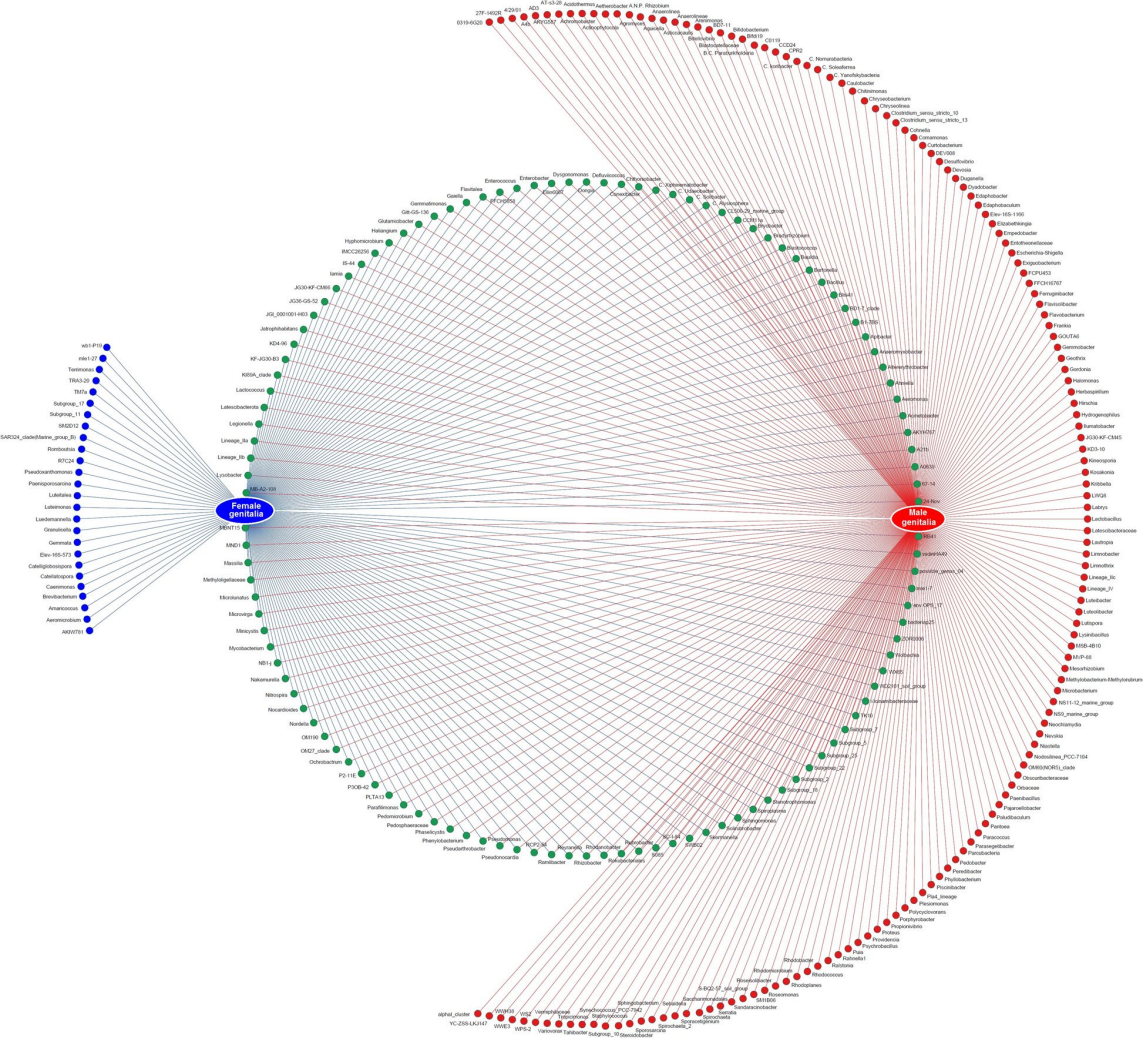
Network analysis showing the shared (green circles crossed by both red/blue lines) and non-shared bacteria of male (red circles) and female (blue circles) genital tract of *Paederus fuscipes*

**Fig. 7:**
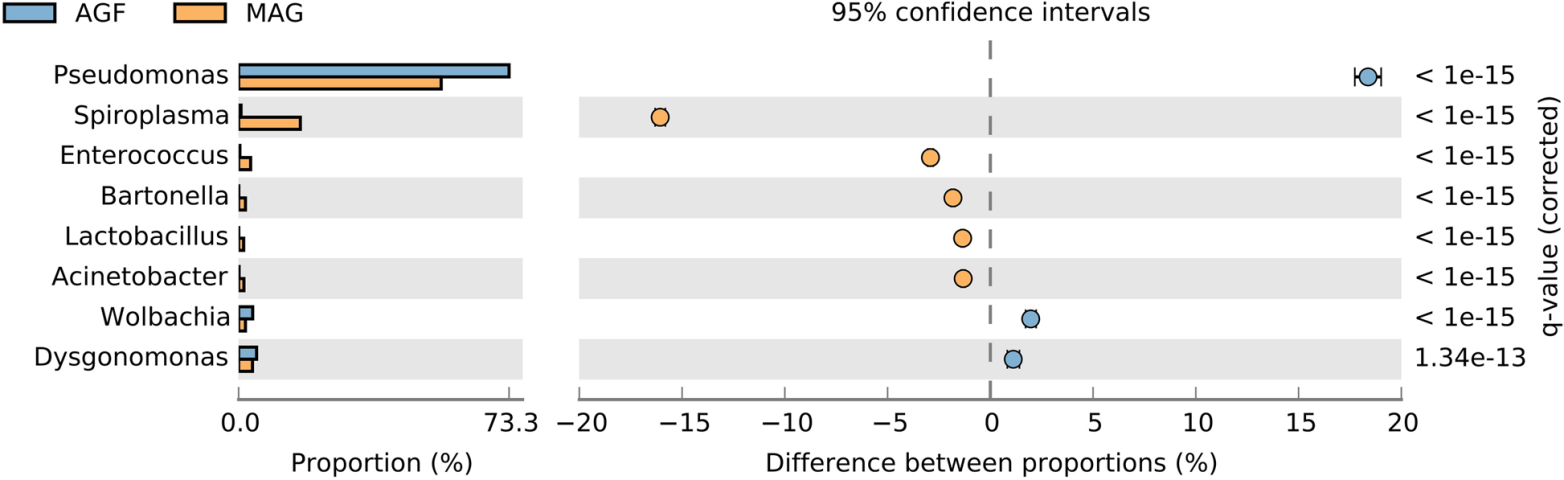
Comparison of genital bacteria profile of male and female *Paederus fuscipes* specimens using STAMP analysis. Comparisons were made at the bacterial genus level. Corrected *p* values were calculated based on Fisher’s exact test method using Storey’s FDR approach. *P* values < 0.05 were taken for comparison. The bar plot indicated in blue or orange shows a positive or negative difference between read proportions. The size effect of >100 reads is included in the comparisons. Differences between samples are shown at 95% confidence intervals

Network analysis of bacteria at the genus level showed that of 298 genera of bacteria known in *P. fuscipes* genitalia, 26 and 153 were exclusively present in female and male genitalia, respectively, and 119 genera were shared between both sexes (Fig. 6). STAMP analysis of reproductive organs of male and female beetles at the genus level demonstrated that *Pseudomonas* is present in greater abundance in females and lesser abundance in males with positive differences, whereas *Spiroplasma* was less abundant in females and more abundant in males with negative differences (Fig. 7). *Wolbachia* / *Spiroplasma* were detected in the genital tissues of male and female beetles with the relative frequencies of 1.67 / 15.17% and 2.30 / 0.56%, respectively. At the species level, *PLPFE* was the most copious bacterium in the genitals of both male and female insects.

### Bacterial communities in the total body of male and female *P. fuscipes*

A total of 759,055 bacterial sequences were obtained from the total body of male (*n* = 365,717) and female (*n* = 393,338) *P. fuscipes*. The sequences identified from the total body of male beetles were classified into 317 families, 468 genera, and 71 species. The identified sequences of the total body of females were also classified into 267 families, 380 genera, and 53 species (Additional file 2: Table S6). Excluding unclassified sequences, the most common OTUs in the total body of both male and female beetles were bacteria belonging to the Pseudomonadaceae.

**Fig. 8:**
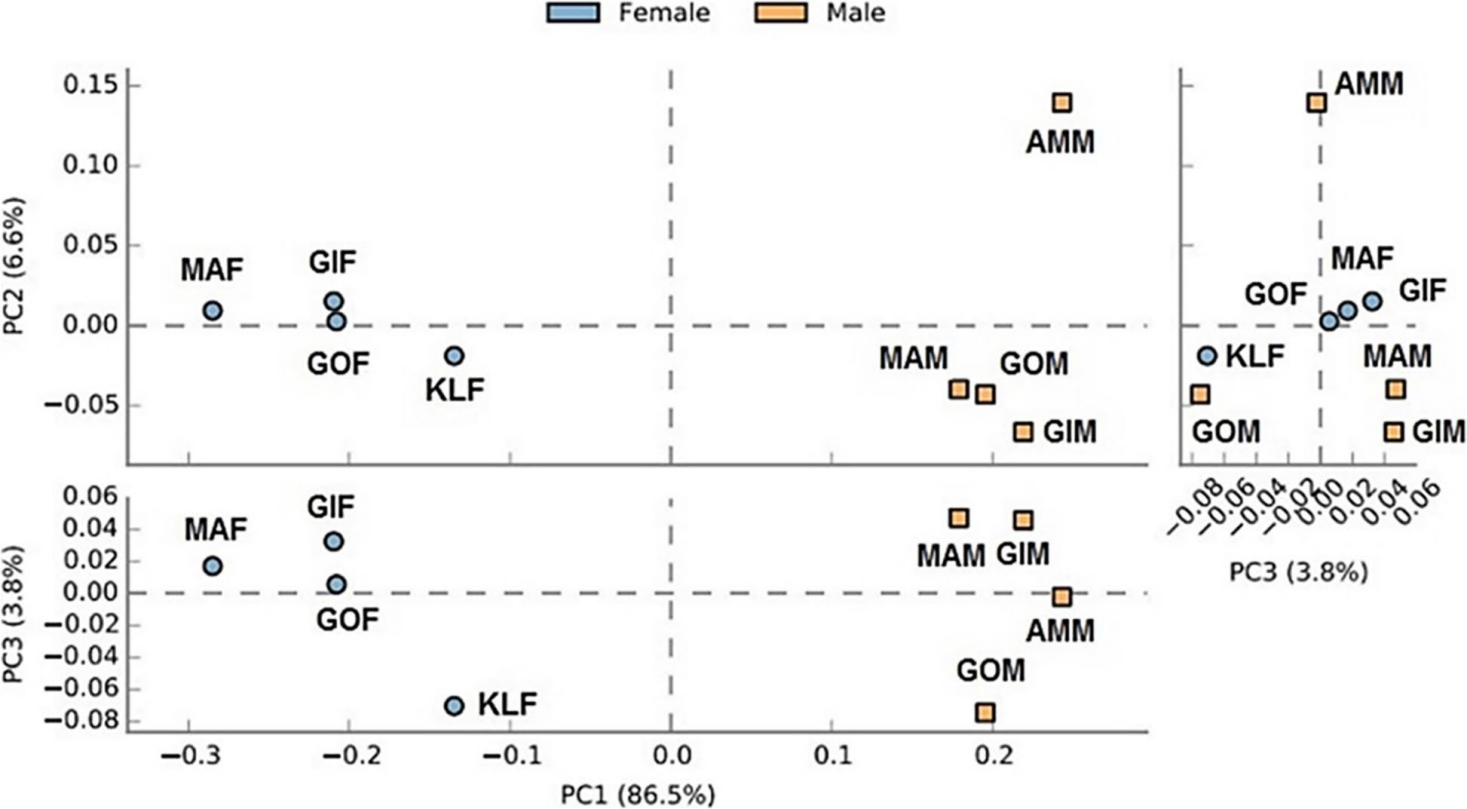
Principal component analysis (PCA) of the abundance of bacterial communities in the total bodies of male and females *Paederus fuscipes*. Bacteria from male (orange squares) beetles are fully grouping separated than female (blue circles) beetles

**Fig. 9:**
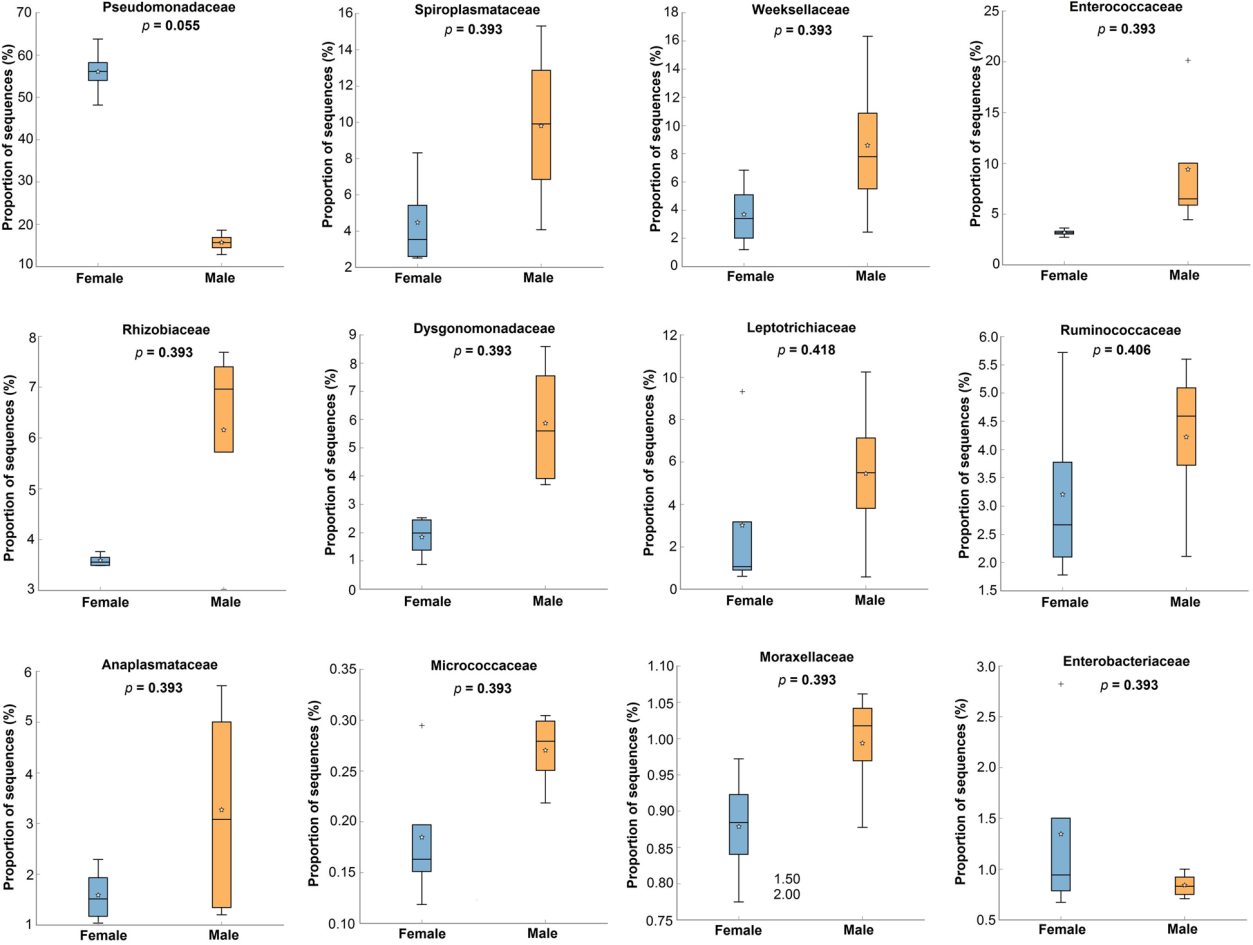
Box plot analysis of 12 bacterial families of Pseudomonadaceae, Spiroplasmataceae, Weeksellaceae, Enterococcaceae, Rhizobiaceae, Dysgonomonadaceae, Leptotrichiaceae, Ruminococcaceae, Anaplasmataceae, Micrococcaceae, Moraxellaceae, and Enterococcaceae with high abundance in the total bodies of female and male *Paederus fuscipes*

The results of PCA analysis for abundance at the family level showed significant differences between the microbiomes of the total bodies of male and female beetles. PCA1 explained 86.5% of the variance (Fig. 8). According to the Welch’s *t*-test, comparing 12 bacterial families with high abundance in the whole body of beetles, Pseudomonadaceae and Enterobacteriaceae were found to be more abundant in females and Anaplasmataceae, Dysgonomonadaceae, Enterococcaceae, Leptotrichiaceae, Micrococcaceae, Moraxellaceae, Rhizobiaceae, Ruminococcaceae, Spiroplasmataceae, and Weeksellaceae in males. However, only Pseudomonadaceae was screened in females with a significant difference from males (56% vs. 16%; *p* <0.055; Fig. 9).

**Fig. 10:**
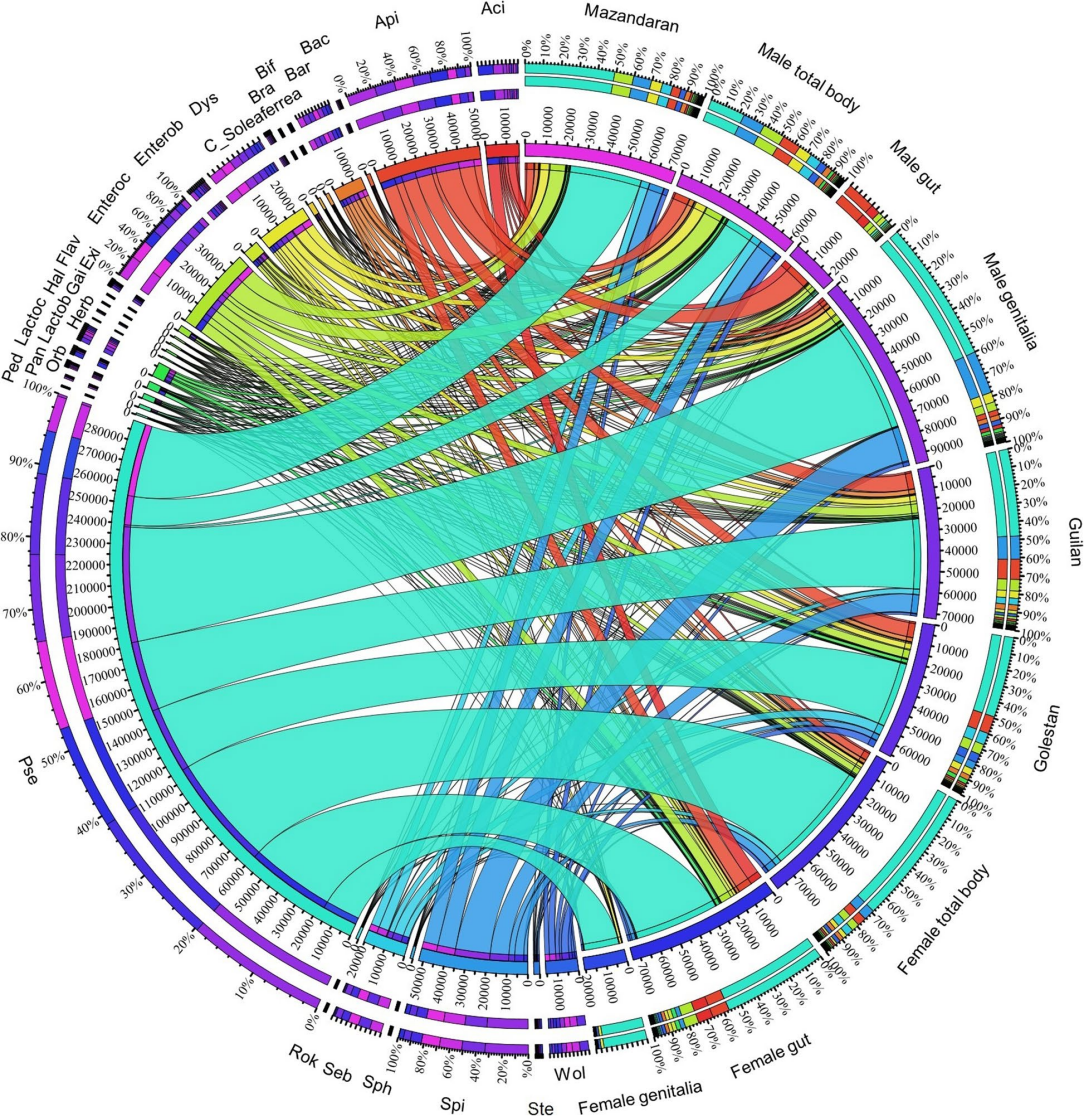
Network analysis representing the shared and exclusive bacterial genera identified from body parts and genders of *Paederus fuscipes* beetles captured from three Southern Caspian Sea Provinces, Guilan, Mazandaran, and Golestan. Only the top 27 bacteria in relative frequencies are shown. Aci, *Acinetobacter*; Api, *Apibacter*; Bac, *Bacillus*; Bar, *Bartonella*; Bif, *Bifidobacterium*; Bra, *Bradyrhizobium; C_Soleaferrea*, *Candidatus_Soleaferrea*; Dys, *Dysgonomonas*; Enterob, *Enterobacter*; Enteroc, *Enterococcus*; Exi, *Exiguobacterium*; Flav, *Flavobacterium*; Gai, *Gaiella*; Hal, *Haliangium*; Herb, *Herbaspirillum*; Lactob, *Lactobacillus*; Lactoc, *Lactococcus*; Orb, *Orbaceae*; Pan, *Pantoea*; Ped, *Pedomicrobium*; Pse, *Pseudomonas*; Rok, *Rokubacteriales*; Seb, *Sebaldella*; Sph, *Sphingomonas*; Spi, *Spiroplasma*; Ste, *Stenotrophomonas*; Wol, *Wolbachia*

At the genus level, 63 genera were exclusively distinguished in the female total bodies, 155 genera in male, and 314 genera were shared between both sexes. Among the shared bacteria, *Pseudomonas*, *Sebaldella*, *Apibacter*, *Enterococcus*, *Wolbachia*, *Spiroplasma*, *Dysgonomonas*, *Lactobacillus*, and *Bartonella* were the nine genera with high abundance (Fig. 10). At the species level, 14 and 25 species were exclusively present in the female and male total bodies, respectively, and 17 species were shared between both sexes (Additional file 2: Table S8).

### Bacterial communities in different body parts of the *P. fuscipes*

**Fig. 11:**
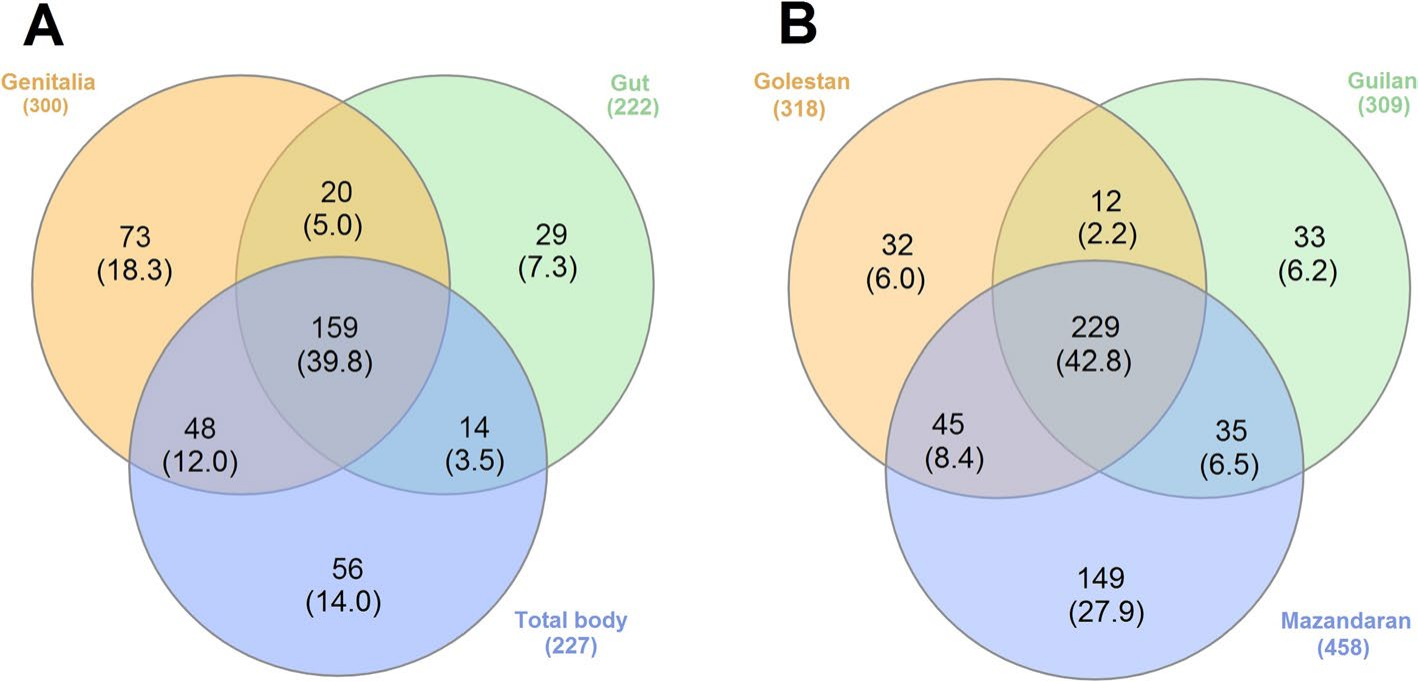
Venn diagram representing the distribution of the bacteria genera across the body parts of the specimens from Mazandaran (left) and Mazandaran-Guilan-Golestan Provinces (right) of the *Paederus fuscipes*. Shared bacteria genera are shown in core. The percentage of bacteria genera for each partner is indicated within parentheses

To understand the distribution/circulation patterns of *P. fuscipes* microbiome, we compared bacteria located in the gut, genitalia, and total body of male with those of female beetles from Mazandaran Province. The distribution of common and unique genera between these compartments is shown in Fig.11A. A total of 159 genera were shared by all studied body parts, 20 genera by gut and genital, 48 genera by genital and the total body, and 14 genera by gut and total body. Also, 29 genera were unique to gut, 73 to genitalia, and 56 to the total body (Fig.11A).

At the species level, three bacteria of *Acinetobacter soli*, *Apibacter adventoris*, and *PLPFE* were shared by all the body parts of male and females, *Scytonema tolypothrichoides*, *Solitalea koreensis*, and *Sphingomonas panni* by male and female total bodies, *Catelliglobosispora koreensis* by female gut and genitalia, *Deinococcus gobiensis* and *Sphingobacterium multivorum* by female gut and male total body, *Flavobacterium ceti*, and *Paenibacillus pectinilyticus* by male genitalia and female total body (Additional file 2: Table S8). Also, three species of *Asticcacaulis biprosthecum*, *Bacillus nealsonii*, and *Nordella oligomobilis* were unique to male genitalia, *Sphingomonas soli* to female genitalia, *Cytophaga hutchinsonii* and *Nocardioides simplex* to male gut, and *Comamonas koreensis*, *Flavobacterium ummariense*, *Sphingobacterium spiritivorum*, and *Stenotrophomonas nitritireducens* to female gut, as well as 15 and 9 bacteria species to the male and female total bodies, respectively (Additional file 2: Table S8).

### Bacterial communities of *P. fuscipes* in the three studied provinces

To understand the effect of environment on the *P. fuscipes* microbiome, we compared bacteria harbored in the specimen of the provinces of Guilan, Golestan, and Mazandaran. As depicted in Fig. 11B, a total of 229 bacteria genera were shared by beetle specimens from the three provinces, 12 genera by Guilan and Golestan, 35 genera by Guilan and Mazandaran, and 45 genera by Golestan and Mazandaran. Also, 33 genera were unique to Guilan, 32 to Golestan, and 149 to total Mazandaran.

At the species level, 11 bacteria *Acinetobacter soli*, *Apibacter adventoris*, *Comamonas aquatic*, *Comamonas sediminis*, *Empedobacter brevis*, *Flavobacterium ceti*, *Lactococcus garvieae*, *PLPFE*, *Rhodococcus corynebacterioides*, *Sebaldella termitidis*, and *Solitalea koreensis* were shared by all the three provinces, *Arenimonas subflava* by Golestan and Guilan, *Acinetobacter baylyi*, *Asticcacaulis biprosthecium*, *Bacillus indicus*, *Comamonas koreensis*, *Nocardioides halotolerans*, and *Sphingomonas panni* by Guilan and Mazandaran, and *Agromyces ramosus*, *Breznakia pachnodae*, *Paenibacillus pectinilyticus*, *Roseomonas terricola*, *Scytonema tolypothrichoides*, and *Sphingobacterium spiritivorum* by Golestan and Mazandaran. Also, five species were unique to Golestan and Guilan and 31 to Mazandaran (Additional file 2: Table S8).

## Discussion

The structure and diversity of microbial communities in Staphylinid materials, despite their key role in the environment and medicine, has received much less attention than that of other arthropods. In this study, for the first time, the microbiomes of the total body, digestive and reproductive tissues of male and female *P. fuscipes* from Southern Caspian littorals were presented through NGS of *16S rRNA* gene. The results showed the high richness and diversity of microbiota associated with *P. fuscipes* beetles, suggesting the persistence of a well-established and conserved core of bacteria. However, interesting disparities were detected between and within the specimens. The results also exhibited a deep divergence between different bacteria, which in fact highlights the specificity of the microbiota and their adaptation to the conditions of the gender and body parts of the rove beetles.

In this study, 99.78% of microorganisms sequenced by metagenomics were bacteria. Among them, Proteobacteria (mainly gamma proteobacteria), Firmicutes, Bacteroidota, Actinobacteriota, and Fusobacteriota were found as the most abundant phyla in *P. fuscipes* population, which is consistent with previous studies on the intestinal microbiotas of soil invertebrate [69–72]. Proteobacteria, as a core taxon and a potential microbial signature of dysbiosis in the guts of the invertebrates, responds to soil contaminants and is closely related to the abundance of antibiotic resistance genes [73]. Likewise, bacterial phyla such as Proteobacteria and Firmicutes can fix atmospheric nitrogen, which is an essential element for the physiological activities of the host including nutrition, growth, and reproduction [74].

Based on the results, *Apibacter*, *Dysgonomonas*, *Enterococcus*, *Pseudomonas*, *Ruminococcus*, *Sebaldella*, *Spiroplasma*, and *Wolbachia* were eight genera accounting for the largest proportions (2–37%) of microorganisms in adult *P. fuscipes* specimens. *Apibacter* spp. are the microaerobic members of the bee gut community that participate in the digestion of monosaccharides and dicarboxylic acids [75]. The genus Dysgonomonas isolated from Isoptera, Coleoptera, and Diptera has been shown to be involved in the breakdown of carbohydrates, such as cellulose and hemicellulose, as well as in the production of vitamin B12 [76–80]. *Enterococcus* has been incriminated in the generation of fecal aggregation pheromone constituents in bombardier beetles [81]. Some *Pseudomonas* species identified in bark beetles are capable of metabolizing the terpenoids, which appear to help the colonization of the host trees by the beetles [82]. *Pseudomonas* species may also aid armyworms in the digestion and detoxification of xenobiotic [83]. In Coleoptera species, the members of the Ruminococcaceae family hydrolyze a set of polysaccharides (such as cellulose) by numerous mechanisms [84]. *Sebaldella* may play a role in supplying nitrogen for host termite and can produce acetic and lactic acids from various sugars [85]. *Spiroplasma* and *Wolbachia*, two reproductive manipulators of arthropods, have shown tissue tropism and asymmetric interactions in the body of *Drosophila melanogaster* [86]. These inherited symbionts are regarded as the guardians of the insect’s immune system and modulate host antimicrobial, antiparasitic, and antiparasitoid responses by direct interacting with humoral immune molecules [17]. Altogether, the OTUs identified from *P. fuscipes* specimens were belonging to 576 genera. In our study, the aforesaid eight bacterial genera were found in high frequency. However, there is no information on their function in providing energy and nutrients and also in regulating the growth, fecundity, and immune responses of *P. fuscipes*, demanding for future investigations to clarify their function in the beetle life history.

Commensal bacteria are mainly obtained from the surrounding environment (and are often reside in the gut) or transmitted directly from the host (via the genitals) to the next generation [7]. Bacteria with both transmission routes are localized in a specific niche within the host body [87, 88]. Therefore, in this study, gut and genital bacteria were studied independently and apart from the total body of the rove beetles. The gut and genitalia of male and female *P. fuscipes* rove beetles harbors a highly conserved core set of bacteria, suggesting that the bacteria are symbiotic. The transmission cycle of many bacteria initiates in the intestinal tract, and the colonization of the reproductive organs allows the vertical transmission of bacteria to the offspring, ensuring their persistence within a population. Although bacterial composition is determined by insect’s diet and its surrounding environment, this study verified the composition of different bacterial species for the specimens in terms of sex and organ. Thus, the data obtained can provide basic insights into the strong characterization and comparison between compartments (Figs. 4, 5, 6, 7, 8, 9, 10, and 11; Additional file 2: Table S8). In addition, intestinal and genital tissues showed a significant divergent of bacteria, the distribution of which reflects microbiota specificity and adaptation to the each tissue. Prominent examples of this distinct adaptation are *Pseudomonas* / *Pseudomonas* as well as *Spiroplasma* / *Apibacter*, which were more abundant in the genitals/guts of the female and male beetles, respectively. These results, in addition to bacteria tissue adaptability, may also imply the interaction between bacteria [43].

Bacterial richness exhibited a decreasing trend in three provinces of Mazandaran, Guilan, and Golestan, respectively (*P* > 0.05). Moreover, bacterial richness of male beetles was higher than that of females and reproductive organs higher than that of guts. In this study, alpha diversity of the bacterial communities associated with *P. fuscipes* was estimated in Qiime using four diversity metrics. Among them, Shannon’s entropy and Faith’s PD seem to be more favorable choices because the former index estimates both richness and evenness in a single equation [89] and the latter index is less sensitive, both to sequencing errors and to errors generated during de novo identification of phylotypes [90].

Our results disclosed that *Pseudomonas* was the dominant genus in the *P. fuscipes* microbiome dataset. Overall, the bacterium was 3 times, 15 times, and 1.3 times more abundant in females than males in the whole body, stomach, and genital, respectively. Even though pederin has weak antibacterial properties [91], herein, we interestingly explored a negative correlation between the relative abundance of pederin-producing *Pseudomonas* (PPP) and microbial diversity, denoting that *Pseudomonas*-rich female beetles have less microbial diversity than their male counterparts, as shown in Fig. 6. This observation presumably confirms the relative activity of PPP in male and female beetles or its interaction with other bacteria, a subject that has also been taken into account in our previous study [43] and requires further studies at the transcriptomics level. In the present study, in addition to PPP, other *Pseudomonas* species were detected in much smaller quantities (Fig. 1); however, their direct and indirect role in pederin synthesis calls to be further investigated.

Intestinal bacterial communities can be used to assess insect feeding habits. Generalist insect species have been shown to possess more diverse microbiota than specialists [92]. The microbiota can hence be expected to vary significantly between oligophagous and polyphagous species or between herbivorous and carnivorous species [93]. The results of the current study signified that the gastrointestinal bacteria of male beetles were significantly less than female beetles in terms of both composition and diversity. These results may simply imply that male *P. fuscipes* are monophagous or oligophagous, and female are polyphagous. The higher diversity of microbiota associated with a polyphagous species can be considered as an adaptive trait, which offers new metabolic potential to expansion diet or tolerate toxic compounds [92, 94]. Further studies, with an enlarged sample size/location or focus on other *Paederus* species, are needed to affirm the pattern observed herein and to precisely elucidate the most influential causes of this divergence.

The studied rove beetles were caught from paddy fields located between the Hyrcanian forests and the southern shores of the Caspian Sea. With very few exceptions, the climates of the study areas are temperate and rainy (without the dry season) with warm summers corresponding to the Cfa of Köppen-Geiger (1930), a climate classification [95]. In terms of diversity indices, no significant difference was observed between the microbiome of the studied specimens in the three provinces (Fig. 2 and Table 2); however, structurally, 43% of bacteria genera were shared among the specimens of three provinces, and the observed differences (Fig. 11B, also see the text) were probably related to the low sample size, as well as the microclimate of the sample collection areas. Based on the mentioned data, in order to establish a better understanding of the circulation pattern of bacteria among *P. fuscipes* populations, we performed bacterial network analysis at the macroclimate (three provinces) level, as well (Fig. 10).

Our study revealed that the body of *P. fuscipes* was a haven for potential human pathogenic bacteria, including *Acinetobacter baylyi*, *Acinetobacter soli*, *Empedobacter brevis*, *Flavobacterium ceti*, *Klebsiella pneumonia*, *Lactococcus garvieae*, *Nordella oligomobilis*, *Prevotella melaninogenica*, *Sphingobacterium spiritivorum*, and *S. thalpophilum*. Once the victims are exposed to the strong pederin toxin, the germs have the ability to cause serious health problems in the host. This idea is reinforced by our other personal observation, suggesting that about 2–3% of beetles are easily crushed while hunting due to their thin cuticle. The mentioned bacteria may be the contributing factor to some local and systemic symptoms in patients with linear dermatitis. The first step in treating linear dermatitis involves the removal of the irritating agent by washing the area with soap and water, which can then be relieved by cool wet soaks and a strong topical steroid [34]. In line with this point, the findings of a study exposed that patients taking oral ciprofloxacin, along with topical steroids, had a statistically faster recovery time than untreated group [33], indicating a concomitant bacterial infection.

The presence of reduced insertion sequence elements and the mosaic-like look of the ped gene clusters in *P. fuscipes* presumably displays insertions and rearrangements in the island and straightforwardly means horizontal gene transfer [44]. Horizontal gene transfer enables bacteria to respond and adapt to their environment much more rapidly by acquiring large DNA sequences from another bacterium in a single transfer [96]. Pathogenic islets are chiefly located in the large and unstable regions of the bacterial genome and can be transmitted to other bacteria by horizontal transfer of the gene [97]. Horizontal gene transfer frequently occurs by conjugation between bacteria with a large taxonomic distance and rarely by conversion and transfer between bacteria of the same or close species [98]. However, in the end, it is the positive and negative selections that determine the fate of preserving or eliminating of these regions in the future [99]. The intestines of soil-dwelling insects provide a hot spot for the transfer of plasmids and transconjugation between bacterial strains [100]. In this study, most of the OTUs in the intestines of male and female beetles were identified as the members of the families Weeksellaceae and Pseudomonadaceae, respectively, which is a confirmation to the primary origin of pederin-producing *Pseudomonas*. A possible source for the horizontal gene transfer of PPP may be Paddy fields, from which beetle specimens were caught and different species of *Pseudomonas* are kept as symbionts in the rhizosphere [101] or as pathogens in the varied parts of the rice plants [102].

The *Apibacter* has recently been described as a novel genus, with several strains from bee species, and it appears to include various insect-associated bacterial species [103, 104]. Comparison of the *Apibacter* genome found in bee guts with their close relatives has shown interesting results. Despite the overall reduction of the *Apibacter* genome, the genes involved in amino acid synthesis and monosaccharide detoxification have been preserved; they have also specifically acquired genes encoding for nitrate respiration, while the genes involved in lipid / histidine breakdown have partially or completely been lost in the genome, and antibiotic resistance genes are sporadically distributed only among *Apibacter* species [105]. The asymmetric distribution of *Apibacter* in the guts of male and female *P. fuscipes* is another emphasis on different feeding habits of rove beetles, presumably evolved in males, toward eating floral parts of plants and metabolism of sugars. However, as noted in the literature, little information is available on the feeding habits of Staphylinids, and more accurate data should be inferred from objective observations in the habitats or from dissecting the insect’s alimentary canal in the laboratory [30].

Interestingly, some OTUs lacked lower level taxonomic identification, which could specify the presence of a number of new species in *P. fuscipes* microbiome. Prominent instances of such OTUs are *Apibacter* (other than *A. adventoris*), *Bartonella*, *Pseudomonas* (other than *PLPFE*), *Sebaldella*, and *Spiroplasma*, and so forth, some of which are being investigated by shotgun sequencing (unpublished data), and some other need to be described in future studies.

It is now generally accepted that microbiome studies require both positive and negative controls [106]. The reason for this view is that the DNAs being studied may be affected by the extraction kits, the chemicals used, and the laboratory environment; thus, the results of the microbiota studies may be biased [107]. The subject is especially important whether the under study specimens contain low microbial biomass (e.g., salivary glands, gut and genital tissues) or include the bulks of polyphagous species with many food connections (e.g., *Paederus* spp.), both of which require more attention. Collectively, the incidence of contaminant bacteria genera (e.g., *Comamonas*, *Deinococcus*, *Sphingomonas*) was minimal in our data. In addition, the species described above have already been presented in metagenomic studies of other insects and have therefore been included in the data analysis.

This study was unique in its kind; it was carried out in relatively wide geographical areas. The read lengths (418 bp) were sufficient for the analysis of OTUs at the species level and showed that the specimens should not be pooled in cases with significant body mass. Another importance of this study basically lies in the discovery of PPP not only in different beetle genera but also in different parts of the beetle’s body. Unequal distribution of PPP in the male and female beetles, as indicated previously [43, 108], is a medical point of view, likely indicating that most cases of dermatitis are caused by female beetles, or at least the wounds caused by them are more severe. However, from the ecological point of view, the different amounts of PPP may have various ecological achievements for male and female beetles, as is the case with blister beetles [109], which requires more extensive studies.

Based on the results of this preliminary study, we suggest research on the microbial dynamics during immature stages of the beetle (i.e., eggs, larvae, and pupae) quantitatively determine the association of the PPP and its metabolites, and examine dermatitis linearis microbiome in patients, and whole genome sequencing of important bacteria associated with *P. fuscipes*. Furthermore, in future research, certain target microbiota should be cultivated, or antibiotics could be used, to remove them from the beetle body to further elucidate their biological functions.

## Conclusions

The present study is a descriptive metagenomic study addressing the theoretical bioecology of *P. fuscipes* through the study of microbiome structure, which requires experimental surveys to elucidate the role of microbiota in the life history of the beetle. The different intestinal and genital microbiomes of male and female *P. fuscipes* reflect the varied bioecology of the beetle genders. A comprehensive overview of *P. fuscipes* microbiome components may eventually lead to ecological insights into the production and utilization of defensive compound of pederin by beetles and humans and also the management of linear dermatitis with the possible use of available antibiotics against bacterial pathogens released by the beetles at the lesion site.

## Supplementary Information

**Additional file 1: Figure S1.** Gastrointestinal and reproductive tissues of micro-dissected male (right) and female (left) *Paederus fuscipes* beetles. *Pseudomonas*-like endosymbionts located at the female accessory glands are responsible for pederin production. **Figure S2.** The aedeagus of representative male *Paederus fuscipes* captured from Khalil-Shahr, Mazandaran. **Figure S3.** The rarefaction curves plotted using Shannon metrics to perceive the species richness within genders, sampling modes, body parts and locations of studied *Paederus fuscipes* specimens.

**Additional file 2: Table S1.** Details of *Paederus fuscipes* rove beetles collected from 23 localities in the three Southern Caspian Sea Provinces, Gilan, Mazandaran, and Golestan, 2019-2020. **Table S2.** Statistics for denoising sequences obtained in this study. **Table S3.** Statistics for merging sequences obtained in this study. **Table S4.** Statistics for filtering sequences obtained in this study. **Table S5.** Statistics for length and counts of sequences obtained in this study. **Table S6.** Overview of total reads and OTUs associated with genders, body parts, and locations of studied *Paederus fuscipes* specimens. **Table S7.** Top five abundance bacteria at the multiple levels of classification in studied *Paederus fuscipes* specimens. **Table S8.** Details of the bacteria species identified from the gut, genitalia, and total bodies of male and female *Paederus fuscipes* beetles captured from three Southern Caspian Sea Provinces, Guilan, Mazandaran, and Golestan, along with isolation sources and bio/ecological importance mentioned in the literature.

## Data Availability

The sequences of *16S rRNA* gene were deposited in the Sequence Read Archive (SRA) at NCBI under BioProject PRJNA852648.

## Abbreviations

*PLPFE*: *Pseudomonas*-like *P. fuscipes* endosymbiont
OTUs: Operational taxonomic units
PPP: Pederin-producing *Pseudomonas*
GOM: Total bodies of pooled male beetles from Golestan province
GOF: Total bodies of pooled female beetles from Golestan province
GIM: Total bodies of pooled male beetles from Guilan province
GIF: Total bodies of pooled female beetles from Guilan province
MAM: Total bodies of pooled male beetles from Mazandaran province
MAF: Total bodies of pooled female beetles from Mazandaran province
AMM: Total body of individual male beetle from Mazandaran province
KLF: Total body of individual female beetle from Mazandaran province
MAG: Genitals of pooled male beetles from Mazandaran province
AGF: Genitals of pooled female beetles from Mazandaran province
MGT: Guts of pooled male beetles from Mazandaran province
FGT: Guts of pooled female beetles from Mazandaran province
