# Supplementary material for: The structure and diversity of microbial communities in Paederus fuscipes (Coleoptera: Staphylinidae): from ecological paradigm to pathobiome
Source: Microbiome. 2023 Jan 20;11:11. doi: 10.1186/s40168-022-01456-z (PMC9862579; doi:10.1186/s40168-022-01456-z)
Supplement: Supplementary file 2 — Additional file 1: Figure S1. Gastrointestinal and reproductive tissues of micro-dissected male (right) and female (left) Paederus fuscipes beetles. Pseudomonas-like endosymbionts located at the female accessory glands are responsible for pederin production. Figure S2. The aedeagus of representative male Paederus fuscipes captured from Khalil-Shahr, Mazandaran. Figure S3. The rarefaction curves plotted using Shannon metrics to perceive the species richness within genders, sampling modes, body parts and locations of studied Paederus fuscipes specimens. [file 40168_2022_1456_MOESM1_ESM.docx]

**
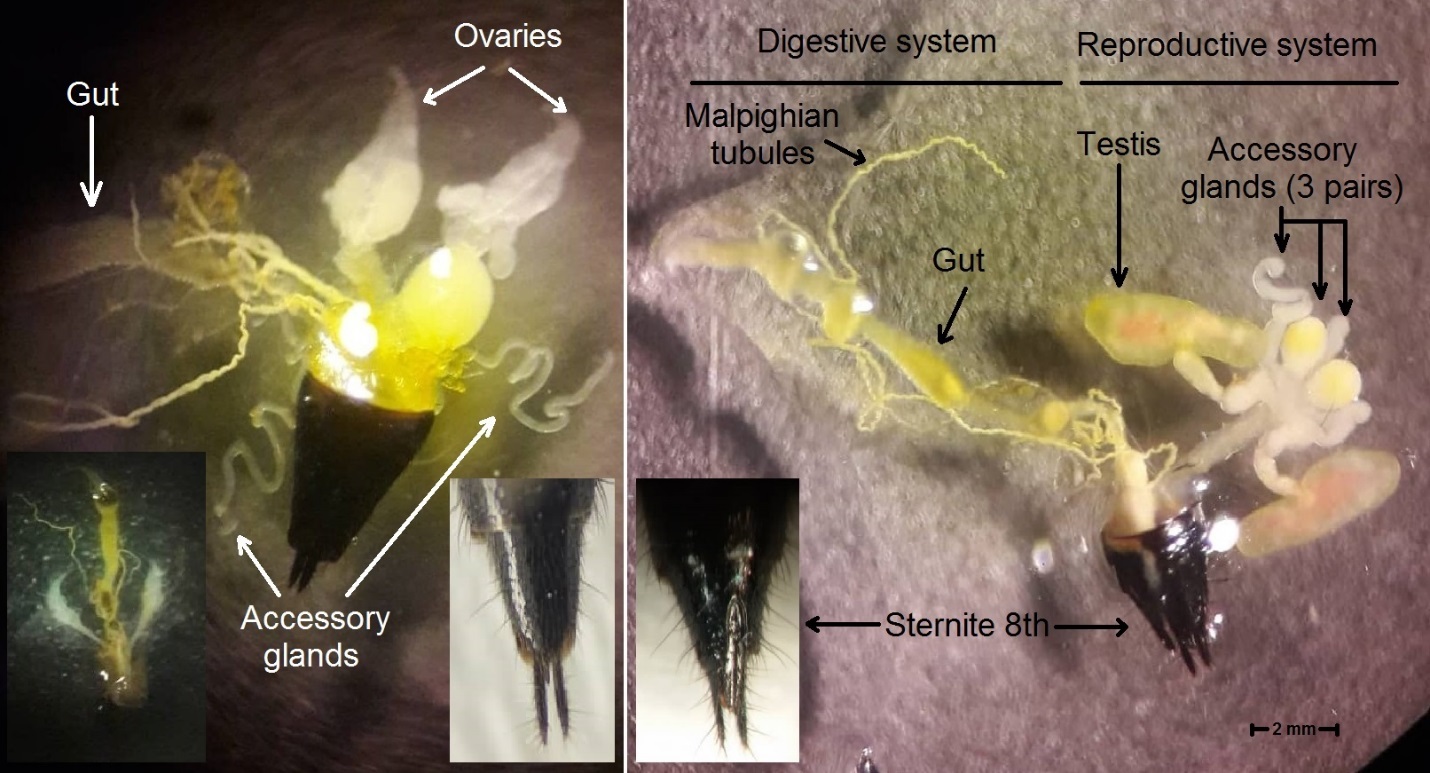
**

**Additional file 1: Figure S 1**: Gastrointestinal and reproductive tissues of micro-dissected male (right) and female (left) *Paederus fuscipes* beetles. *Pseudomonas*-like endosymbionts located at the female accessory glands are responsible for pederin production.


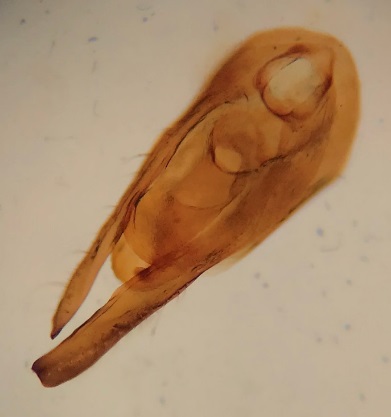


**Additional file 1: Figure S 2**: The aedeagus of representative male *Paederus fuscipes* captured from Khalil-Shahr, Mazandaran


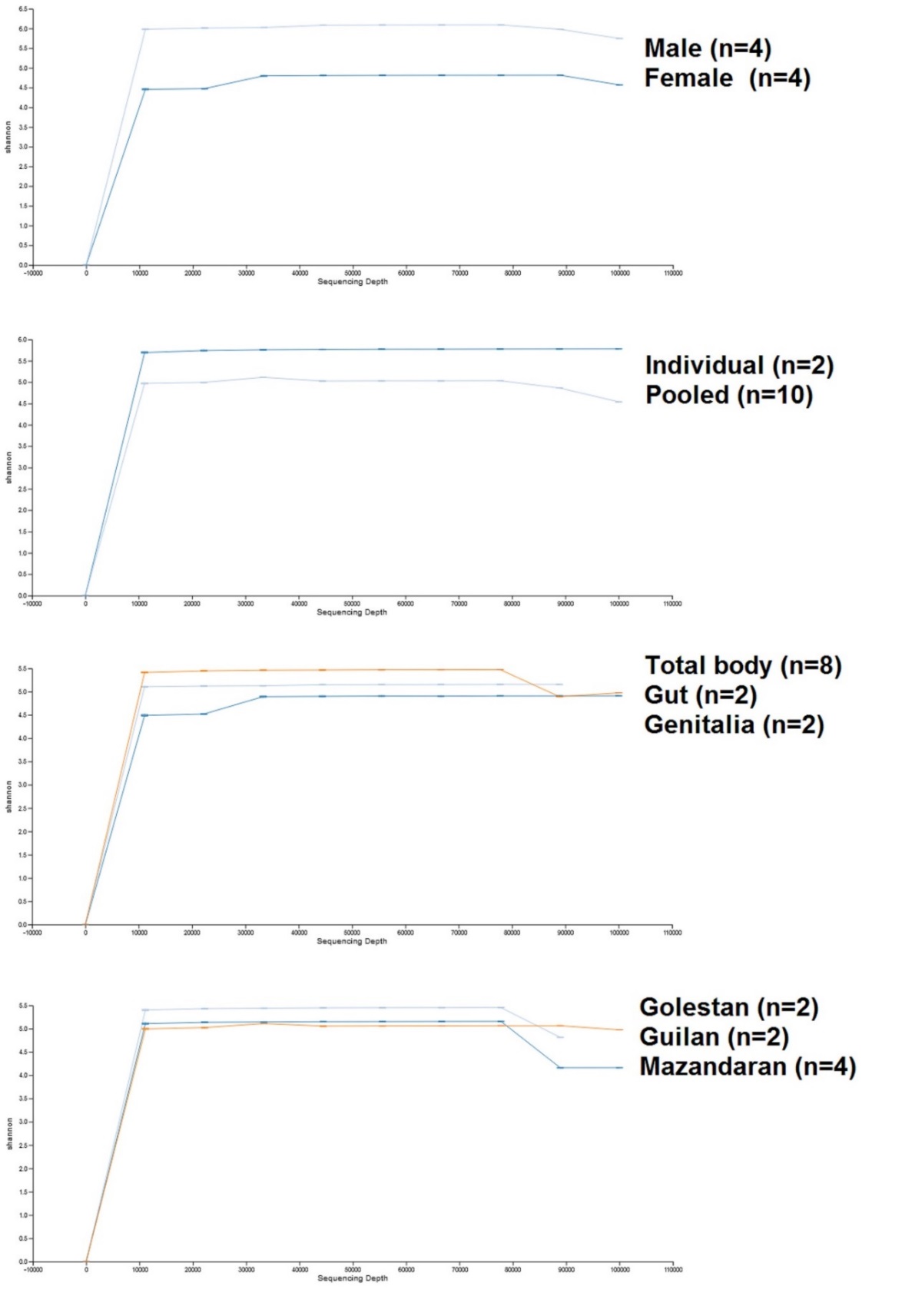


**Additional file 1: Figure S 3**: The rarefaction curves plotted using Shannon metrics to perceive the species richness within genders, sampling modes, body parts and locations of studied ***Paederus fuscipes*** specimens.
