# Supplementary material for: The structure and diversity of microbial communities in Paederus fuscipes (Coleoptera: Staphylinidae): from ecological paradigm to pathobiome
Source: Microbiome. 2023 Jan 20;11:11. doi: 10.1186/s40168-022-01456-z (PMC9862579; doi:10.1186/s40168-022-01456-z)
Supplement: Supplementary file 3 — Additional file 2: Table S1. Details of Paederus fuscipes rove beetles collected from 23 localities in the three Southern Caspian Sea Provinces, Gilan, Mazandaran, and Golestan, 2019-2020. Table S2. Statistics for denoising sequences obtained in this study. Table S3. Statistics for merging sequences obtained in this study. Table S4. Statistics for filtering sequences obtained in this study. Table S5. Statistics for length and counts of sequences obtained in this study. Table S6. Overview of total reads and OTUs associated with genders, body parts, and locations of studied Paederus fuscipes specimens. Table S7. Top five abundance bacteria at the multiple levels of classification in studied Paederus fuscipes specimens. Table S8. Details of the bacteria species identified from the gut, genitalia, and total bodies of male and female Paederus fuscipes beetles captured from three Southern Caspian Sea Provinces, Guilan, Mazandaran, and Golestan, along with isolation sources and bio/ecological importance mentioned in the literature. [file 40168_2022_1456_MOESM2_ESM.docx]

**Additional file 2: Table S1**. Details of *Paederus fuscipes* rove beetles collected from 23 localities in the three Southern Caspian Sea Provinces, Gilan, Mazandaran, and Golestan, 2019-2020.

| **Province** |  | **Location** |  | **Gender** | |  | **Total** | |
| --- | --- | --- | --- | --- | --- | --- | --- | --- |
|  |  |  |  | **Male** | **Female** |  | **Location** | **Province** |
| Golestan |  | Esfahan-Kalateh |  | 11 | 11 |  | 22 | 55 |
|  |  | Bandar-Gaz |  | 14 | 6 |  | 20 |  |
|  |  | Kord-Kuy |  | 4 | 1 |  | 5 |  |
|  |  | Aliabad-Katul |  | 2 | 6 |  | 8 |  |
|  |  |  |  |  |  |  |  |  |
| Mazandaran |  | Sari |  | 0 | 1 |  | 1 | 240 |
|  |  | Khalil-Shahr |  | 11 | 12 |  | 23 |  |
|  |  | Babol |  | 2 | 3 |  | 5 |  |
|  |  | Fereydunkenar |  | 12 | 10 |  | 22 |  |
|  |  | Amol |  | 6+38 | 8+38 |  | 90 |  |
|  |  | Mahmoud-Abad |  | 7 | 7 |  | 14 |  |
|  |  | Nur |  | 10 | 4 |  | 14 |  |
|  |  | Nowshahr |  | 15 | 18 |  | 33 |  |
|  |  | Ramsar |  | 16 | 3 |  | 19 |  |
|  |  | Tonekabon |  | 14 | 5 |  | 19 |  |
|  |  |  |  |  |  |  |  |  |
| Guilan |  | Someh-Sara |  | 8 | 11 |  | 19 | 169 |
|  |  | Langeroud |  | 21 | 6 |  | 27 |  |
|  |  | Bandar Anzali |  | 10 | 6 |  | 16 |  |
|  |  | Rezvanshahr |  | 8 | 13 |  | 21 |  |
|  |  | Lahijan |  | 8 | 6 |  | 14 |  |
|  |  | Khomam |  | 6 | 21 |  | 27 |  |
|  |  | Rasht |  | 7 | 12 |  | 19 |  |
|  |  | Masal |  | 6 | 7 |  | 13 |  |
|  |  | Rudsar |  | 7 | 6 |  | 13 |  |
|  |  |  |  |  |  |  |  |  |
| Total |  |  |  | 243 | 221 |  | 464 | 464 |

**Additional file 2: Table S2**. Statistics for denoising sequences obtained in this study

| **Sample-id** | **Input numeric** | **Filtered numeric** | **Percentage of input passed filter numeric** | **Denoised numeric** | **Non-chimeric numeric** | **Percentage of input non-chimeric numeric** |
| --- | --- | --- | --- | --- | --- | --- |
| MGT | 41151 | 41084 | 99.84 | 40195 | 37417 | 90.93 |
| MAM | 101031 | 100934 | 99.9 | 97393 | 92862 | 91.91 |
| MAG | 120622 | 120463 | 99.87 | 118169 | 113994 | 94.51 |
| MAF | 105173 | 104976 | 99.81 | 102951 | 99607 | 94.71 |
| KLF | 107070 | 106931 | 99.87 | 104137 | 102238 | 95.49 |
| GOM | 94126 | 94020 | 99.89 | 90132 | 87310 | 92.76 |
| GOF | 96494 | 96341 | 99.84 | 93063 | 90227 | 93.51 |
| GIM | 91193 | 91089 | 99.89 | 87606 | 83750 | 91.84 |
| GIF | 106838 | 106672 | 99.84 | 104584 | 101266 | 94.78 |
| FGT | 98893 | 98731 | 99.84 | 96012 | 92717 | 93.75 |
| AMM | 107744 | 107618 | 99.88 | 103835 | 101795 | 94.48 |
| AGF | 29863 | 29791 | 99.76 | 27755 | 27313 | 91.46 |

**Additional file 2: Table S3**. Statistics for merging sequences obtained in this study

| **Sample**  **_name** | **Total**  **_reads** | **Combined**  **_reads** | **Uncombined**  **_reads** | **Percent_**  **combined (%)** | **Combined_base(bp)** | **Min_**  **len(bp)** | **Max_**  **len(bp)** | **Avg_**  **len(bp)** |
| --- | --- | --- | --- | --- | --- | --- | --- | --- |
| AGF | 50,854 | 44,372 | 6,482 | 87.25 | 18,667,245 | 44 | 443 | 421 |
| AMM | 164,051 | 143,158 | 20,893 | 87.26 | 60,276,775 | 44 | 441 | 421 |
| FGT | 157,981 | 138,534 | 19,447 | 87.69 | 58,756,186 | 64 | 441 | 424 |
| GIF | 155,753 | 136,885 | 18,868 | 87.89 | 58,106,811 | 44 | 441 | 424 |
| GIM | 150,560 | 131,919 | 18,641 | 87.62 | 55,442,297 | 44 | 441 | 420 |
| GOF | 154,514 | 135,450 | 19,064 | 87.66 | 57,305,217 | 159 | 441 | 423 |
| GOM | 153,978 | 137,485 | 16,493 | 89.29 | 57,574,937 | 64 | 441 | 419 |
| KLF | 150,501 | 133,602 | 16,899 | 88.77 | 56,293,339 | 44 | 441 | 421 |
| MAF | 151,008 | 134,619 | 16,389 | 89.15 | 57,161,486 | 44 | 441 | 425 |
| MAG | 165,322 | 147,159 | 18,163 | 89.01 | 62,499,752 | 44 | 441 | 425 |
| MAM | 154,023 | 137,349 | 16,674 | 89.17 | 57,729,417 | 64 | 441 | 420 |
| MGT | 54,569 | 52,974 | 1,595 | 97.08 | 22,510,002 | 236 | 441 | 425 |
| #Total | 1,663,114 | 1,473,506 | 189,608 | 88.6 | 622,323,464 | 236 | 441 | 422 |

**Additional file 2: Table S4**. Statistics for filtering sequences obtained in this study

| **Sample Name** | **Raw PE**  **(#)** | **Combined**  **(#)** | **Qualified**  **(#)** | **Nochime**  **(#)** | **Base**  **(nt)** | **AvgLen**  **(nt)** | **Q20** | **Q30** | **GC**  **(%)** | **Effective**  **(%)** |
| --- | --- | --- | --- | --- | --- | --- | --- | --- | --- | --- |
| AGF | 50,854 | 44,372 | 42,684 | 29,863 | 12,596,280 | 422 | 97.67 | 93.21 | 52.89 | 58.72 |
| AMM | 164,051 | 143,158 | 141,099 | 107,744 | 45,450,396 | 422 | 98.4 | 94.88 | 51.86 | 65.68 |
| FGT | 157,981 | 138,534 | 135,221 | 98,893 | 41,991,055 | 425 | 98.16 | 94.34 | 52.02 | 62.6 |
| GIF | 155,753 | 136,885 | 132,662 | 106,838 | 45,372,349 | 425 | 97.96 | 93.86 | 51.68 | 68.59 |
| GIM | 150,560 | 131,919 | 130,128 | 91,193 | 38,402,907 | 421 | 98.43 | 94.91 | 51.3 | 60.57 |
| GOF | 154,514 | 135,450 | 131,410 | 96,494 | 40,939,773 | 424 | 98.05 | 94.07 | 51.9 | 62.45 |
| GOM | 153,978 | 137,485 | 135,703 | 94,126 | 39,440,441 | 419 | 98.53 | 95.18 | 51.64 | 61.13 |
| KLF | 150,501 | 133,602 | 129,937 | 107,070 | 45,132,564 | 422 | 98.14 | 94.26 | 52.05 | 71.14 |
| MAF | 151,008 | 134,619 | 130,037 | 105,173 | 44,722,947 | 425 | 97.95 | 93.79 | 51.58 | 69.65 |
| MAG | 165,322 | 147,159 | 142,686 | 120,622 | 51,272,786 | 425 | 98.09 | 94.09 | 50.97 | 72.96 |
| MAM | 154,023 | 137,349 | 135,561 | 101,031 | 42,546,439 | 421 | 98.47 | 94.99 | 51.04 | 65.59 |
| MGT | 54,569 | 52,974 | 52,468 | 41,151 | 17,491,933 | 425 | 98.46 | 94.9 | 51.74 | 75.41 |

**Additional file 2: Table S5**. Statistics for length and counts of sequences obtained in this study

| **AGF** | |  | **AMM** | |  | **FGT** | |  | **GIF** | |  | **GIM** | |  | **GOF** | |  | **GOM** | |  | **KLF** | |  | **MAF** | |  | **MAG** | |  | **MAM** | |  | **MGT** | |
| --- | --- | --- | --- | --- | --- | --- | --- | --- | --- | --- | --- | --- | --- | --- | --- | --- | --- | --- | --- | --- | --- | --- | --- | --- | --- | --- | --- | --- | --- | --- | --- | --- | --- | --- |
| **LG** | **CT** |  | **LG** | **CT** |  | **LG** | **CT** |  | **LG** | **CT** |  | **LG** | **CT** |  | **LG** | **CT** |  | **LG** | **CT** |  | **LG** | **CT** |  | **LG** | **CT** |  | **LG** | **CT** |  | **LG** | **CT** |  | **LG** | **CT** |
| 324 | 52 |  | 324 | 51 |  | 304 | 104 |  | 309 | 378 |  | 322 | 45 |  | 323 | 58 |  | 323 | 54 |  | 323 | 41 |  | 323 | 43 |  | 323 | 41 |  | 324 | 33 |  | 319 | 13 |
| 334 | 98 |  | 334 | 60 |  | 314 | 214 |  | 319 | 51 |  | 332 | 68 |  | 333 | 74 |  | 333 | 57 |  | 333 | 81 |  | 333 | 78 |  | 333 | 67 |  | 334 | 73 |  | 329 | 16 |
| 344 | 731 |  | 344 | 47 |  | 324 | 52 |  | 329 | 69 |  | 342 | 72 |  | 343 | 73 |  | 343 | 67 |  | 343 | 73 |  | 343 | 88 |  | 343 | 67 |  | 344 | 45 |  | 339 | 16 |
| 354 | 51 |  | 354 | 81 |  | 334 | 85 |  | 339 | 81 |  | 352 | 86 |  | 353 | 243 |  | 353 | 71 |  | 353 | 224 |  | 353 | 271 |  | 353 | 231 |  | 354 | 70 |  | 349 | 14 |
| 364 | 54 |  | 364 | 63 |  | 344 | 105 |  | 349 | 68 |  | 362 | 59 |  | 363 | 73 |  | 363 | 77 |  | 363 | 48 |  | 363 | 28 |  | 363 | 39 |  | 364 | 49 |  | 359 | 13 |
| 374 | 36 |  | 374 | 99 |  | 354 | 203 |  | 359 | 243 |  | 372 | 55 |  | 373 | 61 |  | 373 | 79 |  | 373 | 42 |  | 373 | 26 |  | 373 | 19 |  | 374 | 86 |  | 369 | 10 |
| 384 | 44 |  | 384 | 104 |  | 364 | 51 |  | 369 | 58 |  | 382 | 116 |  | 383 | 81 |  | 383 | 106 |  | 383 | 71 |  | 383 | 51 |  | 383 | 31 |  | 384 | 65 |  | 379 | 15 |
| 394 | 876 |  | 394 | 478 |  | 374 | 35 |  | 379 | 38 |  | 392 | 127 |  | 393 | 127 |  | 393 | 147 |  | 393 | 65 |  | 393 | 65 |  | 393 | 73 |  | 394 | 541 |  | 389 | 13 |
| 404 | 9145 |  | 404 | 40325 |  | 384 | 95 |  | 389 | 42 |  | 402 | 39336 |  | 403 | 27567 |  | 403 | 51859 |  | 403 | 40141 |  | 403 | 20440 |  | 403 | 21532 |  | 404 | 40802 |  | 399 | 4700 |
| 414 | 1519 |  | 414 | 5589 |  | 394 | 314 |  | 399 | 19078 |  | 412 | 2515 |  | 413 | 2221 |  | 413 | 2492 |  | 413 | 1428 |  | 413 | 1065 |  | 413 | 1103 |  | 414 | 6862 |  | 409 | 1323 |
| 424 | 30054 |  | 424 | 94109 |  | 404 | 23126 |  | 409 | 3071 |  | 422 | 87539 |  | 423 | 100761 |  | 423 | 80598 |  | 423 | 87680 |  | 423 | 107841 |  | 423 | 119449 |  | 424 | 86892 |  | 419 | 15941 |
| 434 | 24 |  | 434 | 93 |  | 414 | 3519 |  | 419 | 22232 |  | 432 | 110 |  | 433 | 71 |  | 433 | 96 |  | 433 | 43 |  | 433 | 41 |  | 433 | 34 |  | 434 | 43 |  | 429 | 30391 |
| 444 | 0 |  | 444 | 0 |  | 424 | 107265 |  | 429 | 87240 |  | 442 | 0 |  | 443 | 0 |  | 443 | 0 |  | 443 | 0 |  | 443 | 0 |  | 443 | 0 |  | 444 | 0 |  | 439 | 3 |
|  |  |  |  |  |  | 434 | 53 |  | 439 | 13 |  |  |  |  |  |  |  |  |  |  |  |  |  |  |  |  |  |  |  |  |  |  | 449 | 0 |
|  |  |  |  |  |  | 444 | 0 |  | 449 | 0 |  |  |  |  |  |  |  |  |  |  |  |  |  |  |  |  |  |  |  |  |  |  |  |  |
| ~ 417 | |  | ~ 418 | |  | ~ 420 | |  | ~ 422 | |  | ~ 415 | |  | ~ 418 | |  | ~ 415 | |  | ~ 416 | |  | ~ 419 | |  | ~ 419 | |  | ~ 417 | |  | ~ 423 | |

LG: Length, CT: Count

**Additional file 2: Table S6**. Overview of total reads and OTUs associated with genders, body parts, and locations of studied *Paederus fuscipes* specimens.

|  | | **Sex** | |  | **Body parts** | | | | | | |  | **Locations** | | |  | **All specimens** |
| --- | --- | --- | --- | --- | --- | --- | --- | --- | --- | --- | --- | --- | --- | --- | --- | --- | --- |
|  |  |  |  |  | **Gut** | |  | **Genitalia** | |  | **Total body** |  |  |  |  |  |  |
|  |  | **Male (GOM, GIM, MAM, AMM)** | **Female (GOF, GIF, MAF, KLF)** |  | **Male (MGT)** | **Female (FGT)** |  | **Male (MAG)** | **Female (AGF)** |  | **GOM, GIM, MAM, AMM, GOF, GIF, MAF, KLF** |  | **Guilan (GIM, GIF)** | **Mazandaran**  **(MAM, MAF, AMM, KLF)** | **Golestan (GOM, GOF)** |  | **(GOM,GOF,GIM,GIF,MAM,MAF,AMM,KLF,MAG,AGF,MGT,FGT)** |
| Total reads | | 365,717 | 393,338 |  | 37,417 | 92,717 |  | 113,994 | 27,313 |  | 759,055 |  | 185,016 | 396,502 | 177,537 |  | 1,030,496 |
| No. of OTUs | Phylum | 38 | 32 |  | 16 | 30 |  | 30 | 24 |  | 39 |  | 31 | 35 | 29 |  | 40 |
|  | Class | 99 | 87 |  | 26 | 62 |  | 73 | 49 |  | 106 |  | 75 | 93 | 79 |  | 112 |
|  | Order | 222 | 187 |  | 58 | 131 |  | 161 | 104 |  | 237 |  | 171 | 210 | 179 |  | 249 |
|  | Family | 317 | 267 |  | 87 | 174 |  | 216 | 138 |  | 345 |  | 232 | 311 | 240 |  | 365 |
|  | Genus | 468 | 380 |  | 103 | 203 |  | 278 | 146 |  | 535 |  | 310 | 458 | 318 |  | 576 |
|  | Species | 71 | 53 |  | 10 | 30 |  | 25 | 9 |  | 94 |  | 38 | 69 | 41 |  | 106 |

**Additional file 2: Table S7**. Top five abundance bacteria at the multiple levels of classification in studied *Paederus fuscipes* specimens.

| **Sample ID** | **Phylum (%)** | **Class (%)** | **Order (%)** | **Family (%)** | **Genus (%)** | **Species (%)** |
| --- | --- | --- | --- | --- | --- | --- |
| MGT | Proteobacteria (51.07)  Bacteroidota (33.02)  Firmicutes (13.41)  Actinobacteriota (1.95)  Unclassified (0.19)  Other phyla (0.36) | Gammaproteobacteria (47.91)  Bacteroidia (33.02)  Bacilli (8.98)  Clostridia (4.43)  Alphaproteobacteria (3.15)  Other classes (2.51) | Flavobacteriales (31.28)  Enterobacterales (28.35)  Pseudomonadales (13.11)  Lactobacillales (8.62)  Oscillospirales (4.42)  Other orders (14.22) | Weeksellaceae (31.20)  Enterobacteriaceae (13.85)  Unclassified (10.92)  Moraxellaceae (10.52)  Enterococcaceae (5.86)  Other families (27.65) | Apibacter (31.10)  Unclassified (30.68)  Acinetobacter (10.52)  Enterococcus (5.86)  Enterobacter (4.85)  Other genera (16.99) | Unclassified (69.47)  *Apibacter adventoris* (26.30)  *Acinetobacter soli* (2.41)  PLPFE (1.12)  *Lactococcus garvieae* (0.50)  Other species (0.20) |
|  |  |  |  |  |  |  |
| MAM | Proteobacteria (41.41)  Firmicutes (27.76)  Bacteroidota (14.68)  Actinobacteriota (7.14)  Fusobacteriota (6.08)  Other phyla (2.93) | Gammaproteobacteria (29.19)  Bacilli (21.76)  Bacteroidia (14.67)  Alphaproteobacteria (12.22)  Actinobacteria (6.11)  Other classes (16.05) | Pseudomonadales (19.50)  Entomoplasmatales (15.31)  Rhizobiales (9.72)  Bacteroidales (7.21)  Flavobacteriales (7.18)  Other orders (41.08) | Pseudomonadaceae (18.62)  Spiroplasmataceae (15.31)  Unclassified (10.94)  Rhizobiaceae (7.31)  Dysgonomonadaceae (7.20)  Other families (40.62) | Unclassified (24.44)  Pseudomonas (18.62)  Spiroplasma (15.31)  Dysgonomonas (7.20)  Apibacter (6.52)  Other genera (27.91) | Unclassified (83.85)  PLPFE (12.30)  *Apibacter adventoris* (2.94)  Firmicutes bacterium (0.36)  *Acinetobacter soli* (0.31)  Other species (0.24) |
|  |  |  |  |  |  |  |
| MAG | Proteobacteria (64.84)  Firmicutes (22.00)  Bacteroidota (5.81)  Actinobacteriota (2.85)  Acidobacteriota (0.94)  Other phyla (3.56) | Gammaproteobacteria (56.46)  Bacilli (20.59)  Alphaproteobacteria (8.37)  Bacteroidia (5.81)  Actinobacteria (1.80)  Other classes (6.97) | Pseudomonadales (51.11)  Entomoplasmatales (15.17)  Rhizobiales (5.46)  Lactobacillales (4.43)  Bacteroidales (3.40)  Other orders (20.43) | Pseudomonadaceae (49.81)  Spiroplasmataceae (15.17)  Rhizobiaceae (3.46)  Dysgonomonadaceae (3.40)  Unclassified (3.27)  Other families (24.89) | Pseudomonas (49.81)  Spiroplasma (15.17)  Unclassified (9.23)  Dysgonomonas (3.40)  Enterococcus (2.95)  Other genera (19.44) | Unclassified (52.65)  PLPFE (45.69)  *Apibacter adventoris* (0.91)  *Acinetobacter soli* (0.32)  Firmicutes bacterium (0.19)  Other species (0.24) |
|  |  |  |  |  |  |  |
| MAF | Proteobacteria (75.69)  Firmicutes (12.46)  Bacteroidota (6.16)  Actinobacteriota (3.19)  Fusobacteriota (1.00)  Other phyla (1.50) | Gammaproteobacteria (69.36)  Bacilli (9.74)  Alphaproteobacteria (6.32)  Bacteroidia (6.16)  Clostridia (2.72)  Other classes (5.70) | Pseudomonadales (64.64)  Flavobacteriales (4.55)  Rhizobiales (4.54)  Entomoplasmatales (4.46)  Lactobacillales (4.08)  Other orders (17.73) | Pseudomonadaceae (63.77)  Weeksellaceae (4.50)  Spiroplasmataceae (4.46)  Rhizobiaceae (3.49)  Unclassified (3.30)  Other families (20.48) | Pseudomonas (63.77)  Unclassified (9.89)  Apibacter (4.49)  Spiroplasma (4.46)  Enterococcus (3.20)  Other genera (14.19) | PLPFE (61.49)  Unclassified (35.56)  *Apibacter adventoris* (1.66)  Firmicutes bacterium (0.87)  *Acinetobacter soli* (0.23)  Other species (0.19) |
|  |  |  |  |  |  |  |
| KLF | Proteobacteria (64.42)  Firmicutes (13.88)  Fusobacteriota (9.32)  Actinobacteriota (4.38)  Bacteroidota (2.93)  Other phyla (5.07) | Gammaproteobacteria (56.69)  Alphaproteobacteria (9.73)  Fusobacteriia (9.32)  Bacilli (7.46)  Clostridia (6.41)  Other classes (10.39) | Pseudomonadales (48.97)  Fusobacteriales (9.32)  Rhizobiales (6.55)  Oscillospirales (5.75)  Lactobacillales (3.47)  Other orders (25.95) | Pseudomonadaceae (48.19)  Leptotrichiaceae (9.32)  Ruminococcaceae (5.72)  Unclassified (4.07)  Rhizobiaceae (3.76)  Other families (28.94) | Pseudomonas (48.19)  Unclassified (16.56)  Sebaldella (9.32)  Enterococcus (2.73)  Spiroplasma (2.62)  Other genera (20.58) | Unclassified (51.79)  PLPFE (46.63)  *Apibacter adventoris* (0.68)  Firmicutes bacterium (0.26)  *Acinetobacter soli* (0.24)  Other species (0.40) |
|  |  |  |  |  |  |  |
|  |  |  |  |  |  |  |
| GOM | Proteobacteria (43.34)  Firmicutes (22.69)  Bacteroidota (13.22)  Fusobacteriota (12.25)  Actinobacteriota (6.06)  Other phyla (2.44) | Gammaproteobacteria (26.39)  Alphaproteobacteria (16.95)  Bacilli (15.50)  Bacteroidia (13.20)  Fusobacteriia (10.25)  Other classes (17.71) | Pseudomonadales (17.35)  Lactobacillales (10.31)  Fusobacteriales (10.25)  Rhizobiales (9.93)  Flavobacteriales (9.17)  Other orders (42.99) | Pseudomonadaceae (16.31)  Leptotrichiaceae (10.25)  Weeksellaceae (9.05)  Unclassified (8.28)  Rhizobiaceae (7.69)  Other families (48.42) | Unclassified (24.11)  Pseudomonas (16.31)  Sebaldella (10.25)  Apibacter (9.03)  Enterococcus (6.35)  Other genera (33.95) | Unclassified (77.65)  PLPFE (14.29)  *Apibacter adventoris* (6.74)  Firmicutes bacterium (0.36)  *Acinetobacter soli* (0.32)  Other species (0.64) |
|  |  |  |  |  |  |  |
| GOF | Proteobacteria (71.92)  Bacteroidota (9.65)  Firmicutes (9.63)  Actinobacteriota (4.80)  Acidobacteriota (0.96)  Other phyla (3.04) | Gammaproteobacteria (63.24)  Bacteroidia (9.65)  Alphaproteobacteria (8.68)  Bacilli (7.27)  Actinobacteria (3.76)  Other classes (7.40) | Pseudomonadales (56.91)  Flavobacteriales (6.90)  Rhizobiales (5.28)  Lactobacillales (4.00)  Micrococcales (2.80)  Other orders (24.11) | Pseudomonadaceae (55.93)  Weeksellaceae (6.83)  Unclassified (4.55)  Rhizobiaceae (3.61)  Enterococcaceae (3.15)  Other families (25.93) | Pseudomonas (55.93)  Unclassified (12.76)  Apibacter (6.82)  Enterococcus (3.15)  Dysgonomonas (2.52)  Other genera (18.82) | PLPFE (53.82)  Unclassified (40.64)  *Apibacter adventoris* (4.74)  Firmicutes bacterium (0.29)  *Acinetobacter soli* (0.27)  Other species (0.24) |
|  |  |  |  |  |  |  |
| GIM | Proteobacteria (34.75)  Firmicutes (28.08)  Bacteroidota (20.75)  Actinobacteriota (6.83)  Fusobacteriota (4.89)  Other phyla (4.70) | Gammaproteobacteria (23.07)  Bacilli (22.00)  Bacteroidia (20.74)  Alphaproteobacteria (11.68)  Clostridia (6.08)  Other classes (16.43) | Flavobacteriales (16.44)  Pseudomonadales (16.05)  Entomoplasmatales (12.05)  Rhizobiales (9.10)  Lactobacillales (7.92)  Other orders (38.44) | Weeksellaceae (16.32)  Pseudomonadaceae (14.99)  Spiroplasmataceae (12.05)  Enterococcaceae (6.64)  Rhizobiaceae (6.61)  Other families (43.39) | Unclassified (19.88)  Apibacter (16.28)  Pseudomonas (14.99)  Spiroplasma (12.05)  Enterococcus (6.64)  Other genera (30.16) | Unclassified (76.52)  PLPFE (12.64)  *Apibacter adventoris* (8.95)  Firmicutes bacterium (1.17)  *Acinetobacter soli* (0.31)  Other species (0.41) |
|  |  |  |  |  |  |  |
| GIF | Proteobacteria (70.52)  Firmicutes (18.89)  Bacteroidota (4.89)  Actinobacteriota (3.22)  Fusobacteriota (1.13)  Other phyla (1.35) | Gammaproteobacteria (64.32)  Bacilli (15.19)  Alphaproteobacteria (6.21)  Bacteroidia (4.88)  Clostridia (3.70)  Other classes (5.70) | Pseudomonadales (27.62)  Entomoplasmatales (8.31)  Rhizobiales (4.65)  Lactobacillales (4.45)  Enterobacterales (4.17)  Other orders (50.80) | Pseudomonadaceae (56.34)  Spiroplasmataceae (8.31)  Unclassified (3.72)  Enterococcaceae (3.63)  Rhizobiaceae (3.50)  Other families (24.50) | Pseudomonas (56.34)  Unclassified (11.13)  Spiroplasma (8.31)  Enterococcus (3.63)  Dysgonomonas (2.43)  Other genera (18.16) | PLPFE (54.47)  Unclassified (43.59)  *Apibacter adventoris* (1.19)  Firmicutes bacterium (0.31)  *Acinetobacter soli* (0.24)  Other species (0.20) |
|  |  |  |  |  |  |  |
| FGT | Proteobacteria (68.88)  Firmicutes (13.45)  Bacteroidota (9.70)  Actinobacteriota (4.55)  Acidobacteriota (0.96)  Other phyla (2.46) | Gammaproteobacteria (56.56)  Bacilli (10.31)  Bacteroidia (8.87)  Alphaproteobacteria (7.10)  Actinobacteria (3.73)  Other classes (13.43) | Pseudomonadales (51.82)  Lactobacillales (8.52)  Flavobacteriales (7.75)  Lactobacillales (8.52)  Enterobacterales (5.21)  Other orders (18.18) | Pseudomonadaceae (45.31)  Weeksellaceae (7.66)  Moraxellaceae (6.55)  Enterococcaceae (5.93)  Unclassified (4.33)  Other families (30.22) | Pseudomonas (45.31)  Unclassified (13.68)  Apibacter (7.55)  Acinetobacter (6.55)  Enterococcus (5.93)  Other genera (20.98) | Unclassified (51.27)  PLPFE (43.00)  *Apibacter adventoris* (2.49)  *Acinetobacter soli* (1.86)  *Lactococcus garvieae* (0.82)  Other species (0.56) |
|  |  |  |  |  |  |  |
|  |  |  |  |  |  |  |
| AMM | Proteobacteria (37.58)  Firmicutes (33.71)  Bacteroidota (13.52)  Actinobacteriota (8.47)  Acidobacteriota (1.37)  Other phyla (5.35) | Bacilli (30.90)  Gammaproteobacteria (23.09)  Alphaproteobacteria (14.48)  Bacteroidia (13.30)  Actinobacteria (6.36)  Other classes (11.87) | Lactobacillales (21.76)  Pseudomonadales (13.86)  Bacteroidales (8.59)  Entomoplasmatales (7.80)  Rhizobiales (7.04)  Other orders (40.95) | Enterococcaceae (20.16)  Pseudomonadaceae (12.86)  Dysgonomonadaceae (8.59)  Spiroplasmataceae (7.78)  Unclassified (5.18)  Other families (45.43) | Enterococcus (20.16)  Unclassified (16.76)  Pseudomonas (12.86)  Dysgonomonas (8.59)  Spiroplasma (7.78)  Other genera (33.85) | Unclassified (85.00)  PLPFE (11.80)  *Apibacter adventoris* (1.37)  Candidatus *Fonsibacter* (0.67)  Firmicutes bacterium (0.32)  Other species (0.84) |
|  |  |  |  |  |  |  |
| AGF | Proteobacteria (73.24)  Unclassified (7.71)  Actinobacteriota (6.11)  Bacteroidota (5.43)  Acidobacteriota (2.33)  Other phyla (5.18) | Gammaproteobacteria (66.40)  Unclassified (7.75)  Alphaproteobacteria (6.84)  Bacteroidia (5.43)  Thermoleophilia (2.36)  Other classes (11.22) | Pseudomonadales (63.72)  Unclassified (8.05)  Bacteroidales (4.22)  Rickettsiales (3.32)  Rhizobiales (2.25)  Other orders (18.44) | Pseudomonadaceae (63.62)  Unclassified (10.03)  Dysgonomonadaceae (4.22)  Anaplasmataceae (3.30)  Weeksellaceae (1.06)  Other families (17.77) | Pseudomonas (63.62)  Unclassified (13.16)  Dysgonomonas (4.22)  Wolbachia (3.30)  Apibacter (0.10)  Other genera (15.06) | PLPFE (63.56)  Unclassified (35.15)  *Apibacter adventoris* (1.06)  *Catelliglobosispora koreensis* (0.13)  *Sphingomonas soli* (0.03)  Other species (0.07) |

* PLPFE: *Pseudomonas‐like P. fuscipes* endosymbiont

**Additional file 2: Table S8**. Details of the bacteria species identified from the gut, genitalia, and total bodies of male and female *Paederus fuscipes* beetles captured from three Southern Caspian Sea Provinces, Guilan, Mazandaran, and Golestan, along with isolation sources and bio/ecological importance mentioned in the literature.

| **Species** | **Isolation source in current study** | | | | | | | | | **Representative isolation sources in the literature** | **Bio/ecological significance** |
| --- | --- | --- | --- | --- | --- | --- | --- | --- | --- | --- | --- |
|  | **Guilan** | **Mazandaran** | **Golestan** | **Female gut** | **Male gut** | **Female genitalia** | **Male genitalia** | **Female** **total** **body** | **Male total body** |  |  |
| *Acinetobacter antiviralis* |  | X |  |  |  |  |  |  | X | Tobacco plant roots [1] | Inhibitory effects on Tobacco mosaic virus [1] |
| *Acinetobacter baylyi* | X | X |  | X | X |  | X |  | X | As a model/soil organism [2]; as a potential  human opportunistic pathogen [3] | Ability to obtain exogenous DNA from the environment and integrate it into chromosomal DNA [2]; can cut down lignin derived monoaromatic compounds [4] |
| *Acinetobacter soli* | X | X | X | X | X | X | X | X | X | Forest soil, vegetables, livestock products [5] sandfly gut [6] | Maybe as a soil fertilizer [7], as a neonatal pathogen in ICU [8] |
| *Actinocorallia longicatena* |  |  | X |  |  |  |  | X |  | A related species was isolated from the head of an ant, *Lasius*  *fuliginosus* [9] | An actinomycete forming sporophores resembling coral [10] |
| *Agromyces ramosus* |  | X | X | X |  |  |  | X | X | In large numbers in many soils [11] | As predator of various gram-positive and gram-negative soil bacteria [11] |
| *Apibacter adventoris* | X | X | X | X | X | X | X | X | X | Honey/ bumble bees guts [12] | Apibacter species utilize microaerobic respiration and fermentation to breakdown monosaccharides and dicarboxylic acids.  They also encode a type IX secretion system and Rhs or VgrG proteins used in intercellular communications.  They are adapted to living in the gut environment [12] |
| *Arenimonas subflava* | X |  | X |  |  |  |  | X |  | A drinking water network [13] | Unknown |
| *Asticcacaulis biprosthecium* | X | X |  |  |  |  | X | X |  | Freshwater [14] | Unknown |
| *Bacillus halmapalus* |  | X |  |  |  |  |  | X |  | Termite gut [15]; decomposing reed rhizomes [16]; marine environment [17] | An alkaliphilic and alkalitolerant bacterium [15, 16] |
| *Bacillus indicus* | X | X |  | X |  |  | X | X |  | Aquifer [18];  soil, water and the gastro-intestinal tract of animals/humans [19] | As food probiotics [20]; containing carbohydrate active enzymes [19] |
| *Bacillus nealsonii* |  | X |  |  |  |  | X |  |  | a spacecraft-assembly facility [21]; lake sediment [22] | The bacterium capable reducing aqueous mercury [23]; and production of  surfactin-like biosurfactants for soil remediation; Its spores are resistance to UV, gamma rays, H2O2 and desiccation [21] |
| *Bacillus psychrosaccharolyticus* |  |  | X |  |  |  |  |  | X | soil or lowland marsh [24] | A psychrophilic/saccharolyticus bacterium that is a potential source of 2′-deoxyribosyltransferase for large scale nucleoside synthesis [24] |
| *Bacillus thermoamylovorans* |  | X |  |  |  |  |  | X |  | palm wine [25];  a hot spring [26] | a moderately thermophilic  and amylolytic bacterium [25];  as a contaminant of food ingredients and raw milk, but also of sterilised milk and dairy products [27] |
| *Brevundimonas bacteroides* |  |  | X |  |  |  |  |  | X | Members of the genus are found in diverse environments, from soil [28] and sediment [29]to human [30] | As a growth promoter in agriculture [31], bioremediation tool [32] and opportunistic pathogens [33]. Some species produce melanin-like pigments offering protection to the cells against UVC exposure [34] |
| *Breznakia pachnodae* |  | X | X |  |  |  |  |  | X | gut of the scarab beetle larva, Pachnoda ephippiata [35] | An obligatory anaerobic fermenting bacterium [35] |
| *Caballeronia glathei* |  | X |  |  |  |  | X | X | X | Diverse ecosystems such as soils, plants, and even animal/human bodies [36] | As a plant beneficial bacterium [36]; The strain DSM50014 contains a complete iac (indole-3-acetic acid catabolism) gene cluster and is capable to utilize IAA as a sole source of carbon and energy [37] |
| *Carnobacterium maltaromaticum* |  | X |  |  |  |  |  |  | X | Frequently isolated f  rom natural environments and foods [38] | While other species are suggested as probiotic in aquaculture, C. maltaromaticum can be a fish pathogen [38] |
| *Catelliglobosispora koreensis* |  | X |  | X |  | X |  |  |  | soil from a gold mine cave [39] | Unknown |
| *Chitinilyticum litopenaei* |  |  | X |  |  |  |  | X |  | Freshwater used for culture of Pacific white shrimp, Litopenaeus vannamei [40] | With strong chitinolytic activity, exclusively utilizes chitin as the carbon, nitrogen and energy source [40] |
| *Comamonas aquatica* | X | X | X | X |  |  | X | X | X | [Human and environmental specimens](https://www.sciencedirect.com/topics/medicine-and-dentistry/human-specimen) [41] | The strain D1877 accelerates development of Caenorhabditis elegans [42] |
| *Comamonas koreensis* | X | X |  | X |  |  |  |  | X | a wetland sample [43] | An iron-reducing bacterium [44] |
| *Comamonas odontotermitis* |  | X |  |  |  |  |  |  | X | Gut of the fungus-growing termite Odontotermes formosanus [45] | An important bacterium that breaks down glyphosate [46] |
| *Comamonas sediminis* | X | X | X | X |  |  |  | X | X | Lagoon sediments [47] | A 4-chlorobenzoate-degrading strain [48] |
| *Cytophaga hutchinsonii* |  | X |  |  | X |  |  |  |  | Soil [49] | Able to efficiently digest cellulose and glide quickly along solid surfaces [50] |
| *Deinococcus gobiensis* |  | X |  | X |  |  |  |  | X | Sand sample [51]) | Extremely resistant to radiation [51] |
| *Empedobacter brevis* | X | X | X | X | X |  | X | X | X | Water sources, plants, soils, and hospital environments [52] | Involved in nosocomial sepsis, pneumonia, and cutaneous infections [52]; inseticidal activities against rice Lepidioptera pests [53] |
| *Enterococcus cecorum* |  | X |  |  |  |  |  | X |  | As a commensal in the intestines of mammals and birds and emerging pathogen in aviculture [54] | Pathogenic and commensal isolates from different sources are bio/ecologically different [55] |
| *Flavobacterium ceti* | X | X | X |  |  |  | X | X | X | Lung and liver of beaked whales [56]; human clinical samples [57] | Unknown |
| *Flavobacterium indicum* |  | X |  |  |  |  |  |  | X | Warm spring water [58] | Able to degrade some macromolecules for instance gelatin, casein, and starch [59] |
| *Flavobacterium ummariense* |  | X |  | X |  |  |  |  |  | Hexachlorocyclohexane-contaminated soil [60] | Probably soil bioremediation  [60] |
| *Hymenobacter arcticus* | X |  |  |  |  |  |  |  | X | Glacial till [61] | Unknown |
| *Klebsiella pneumoniae* |  | X |  |  |  |  |  | X |  | Normal flora of the soil, plants endosymbionts, vertebrate mouth, skin, and intestines [62] | As a biofertilizer by [fixing nitrogen](https://en.wikipedia.org/wiki/Nitrogen_fixation) in anaerobic conditions [63];  common causes of pneumonia, urinary tract and bloodstream infections [62] |
| *Lactococcus garvieae* | X | X | X | X | X |  | X | X | X | Guts of *Apis nigrocincta* [64], *Anabrus simplex* [65],  *Musca domestica*  [66], and Harpalus pensylvanicus [67] | As a well-known fish pathogen and emergent disease agent in human [24] |
| *Mesorhizobium albiziae* |  | X |  |  |  |  |  | X |  | Root nodule of leguminous Albizia kalkora [68] | As a legume growth-promoting rhizobium and highly acid sensitive strain [69] |
| *Nocardioides halotolerans* | X | X |  | X |  |  |  | X | X | Farming field soil [70] | A halotolerant actinobacterium [70] |
| *Nocardioides simplex* |  | X |  |  | X |  |  |  |  | Rice soil [71] | Active in sterol catabolism [72] |
| *Nordella oligomobilis* |  | X |  |  |  |  | X |  |  | Epilithic biofilm sample [73] | An amoeba-associated  Microorganism may cases pneumonia [74] |
| *Oscillatoriales cyanobacterium* | X |  |  |  |  |  |  |  | X | Tibetan lacustrine sediment [75] | Oscillatoriales accounts for 60% of all secondary metabolites isolated from marine cyanobacterial [76] |
| *Pseudomonas‐like Paederus fuscipes endosymbiont* | X | X | X | X | X | X | X | X | X | Paederus fuscipes  [77] | See the text |
| *Paenibacillus crassostreae* | X |  |  |  |  |  |  |  | X | Pacific oyster, *Crassostrea gigas* [78] | Unknown |
| *Paenibacillus pectinilyticus* |  | X | X |  |  |  | X | X | X | Gut of orthopteran *Diestrammena apicalis* [79] | Ability to break down pectin [79] |
| *Peredibacter starrii* |  | X |  |  |  |  |  |  | X | Soil [80] | Unknown |
| *Phormidium chlorinum* |  | X |  |  |  |  |  | X |  | Soil [81] | Unknown |
| *Polynucleobacter cosmopolitanus* |  | X |  |  |  |  |  |  | X | A cosmopolitan species mainly found in freshwater, and oligosaline habitats, but absent from eusaline and hypersaline environments [82] | Unknown |
| *Prevotella melaninogenica* | X |  |  |  |  |  |  |  | X | As commensal anaerobic microbiota of the oropharynx, colon, and vagina where it can be opportunistic pathogen [83] | Carbohydrate metabolism [84] |
| *Pseudonocardia hispaniensis* |  | X |  |  |  |  |  |  | X | Industrial wastewater [85] | The genus has an important role in production of secondary metabolites [86] |
| *Psychrobacter meningitidis* | X |  |  |  |  |  |  | X |  | Vegetables [87]; cerebrospinal fluid [88] | Producing pectic enzymes [87] |
| *Quadrisphaera granulorum* |  |  | X |  |  |  |  |  | X | Aerobic granules [89] | A recent innovation in biological wastewater treatment [89] |
| *Rhodococcus coprophilus* |  | X |  |  |  |  |  |  | X | Commonly in herbivore dung and aquatic habitats [90] | As a particular marker organism of fecal pollution [91] |
| *Rhodococcus corynebacterioides* | X | X | X |  |  |  | X | X | X | Genitalia of male (OM243868) and abdomen of female (OM243853) *Philaenus spumarius* | As endophytic bacterium in plant as well as the cause of various infections in humans [92] |
| *Rhodococcus jostii* |  | X |  |  |  |  |  |  | X | Soil [93] | Lignin degradation [93] |
| *Roseomonas terricola* |  | X | X |  |  |  |  |  | X | agricultural soil [94] | Unknown |
| *Scytonema tolypothrichoides* |  | X | X |  |  |  |  | X | X | Mainly as biofilms in subaerial habitats (e.g. on stone and mortar monuments and sculptures;  [95]) | Due to its hydrophobic nature, it grows well on solid substrates [95] |
| *Sebaldella termitidis* | X | X | X | X |  |  |  | X | X | Termite intestine [96] | Providing nitrogen to the termite host [96] |
| *Solitalea koreensis* | X | X | X |  |  |  |  | X | X | Greenhouse soil [97] | Unknown |
| *Sphingobacterium multivorum* |  | X |  | X |  |  |  |  | X | Wheat straw [98] | Lignocellulose degradation [98] |
| *Sphingobacterium spiritivorum* |  | X | X | X |  |  |  | X |  | Ubiquitous in natural environments, however may found in human clinical specimens including vaginal secretions, sputum etc. [99] | Intrinsically resistant to  Many antimicrobials; may cause opportunistic human infections [100, 101] |
| *Sphingobacterium thalpophilum* |  | X |  |  |  |  |  | X |  | variety of infections, including peritonitis, wound infections and abscesses [41] | Cause opportunistic human infections [101] |
| *Sphingobacterium thermophilum* |  | X |  |  |  |  |  |  |  | Compost [102] | Unknown |
| *Sphingomonas panni* | X | X |  |  |  |  |  | X | X | A sponge in the medical clinic for small animals and ungulates [103] | Unknown |
| *Sphingomonas soli* |  | X |  |  |  | X |  |  |  | Soil [104] | A β-glucosidase-producing bacterium [104] |
| *Stenotrophomonas nitritireducens* |  | X |  | X |  |  |  |  |  | Soil [105] | Ability to reduce [nitrite](https://en.wikipedia.org/wiki/Nitrite) [105] |
| *Stenotrophomonas rhizophila* |  | X |  |  |  |  |  |  | X | A plant-associated bacterium [106] | Antifungal activity  against phytopathogenic and human-pathogenic fungi [106] |
| *Thorsellia anophelis* |  | X |  |  |  |  |  |  | X | midgut of the mosquito Anopheles arabiensis [107] | As a potential weapon in the fight against malaria [108] |
| *Thorsellia kandunguensis* |  | X |  |  |  |  |  |  | X | larvae of Anopheles arabiensis [109] | As a potential weapon in the fight against malaria [108] |

OM243868, OM243853: accession numbers from genebank

1.

**References for literature cited in Additional file 2: Table S8**

1. Lee J-S, Lee K-C, Kim K-K, Hwang I-C, Jang C, Kim N-G, Yeo W-H, Kim B-S, Yu Y-M, Ahn J-S, biotechnology: *Acinetobacter antiviralis* sp. nov., from tobacco plant roots. J Microbiol. 2009, 19:250-256.

2. Elliott KT, Neidle EL: *Acinetobacter baylyi* ADP1: transforming the choice of model organism. IUBMB Life. 2011, 63:1075-1080.

3. Chen T-L, Siu L-K, Lee Y-T, Chen C-P, Huang L-Y, Wu RC-C, Cho W-L, Fung C-P: *Acinetobacter baylyi* as a pathogen for opportunistic infection. J Clin Microbiol. 2008, 46:2938-2944.

4. Phale PS, Malhotra H, Shah BA: Degradation strategies and associated regulatory mechanisms/features for aromatic compound metabolism in bacteria. Adv Appl Microbiol. 2020, 112:1-65.

5. Al Atrouni A, Joly-Guillou M-L, Hamze M, Kempf M: Reservoirs of Non-baumannii *Acinetobacter* species. Front Microbiol. 2016, 7.

6. Maleki-Ravasan N, Oshaghi MA, Afshar D, Arandian MH, Hajikhani S, Akhavan AA, Yakhchali B, Shirazi MH, Rassi Y, Jafari R, et al: Aerobic bacterial flora of biotic and abiotic compartments of a hyperendemic Zoonotic Cutaneous Leishmaniasis (ZCL) focus. Parasites Vectors. 2015, 8:1-22.

7. Kim D, Baik KS, Kim MS, Park SC, Kim SS, Rhee MS, Kwak YS, Seong CN: Acinetobacter soli sp. nov., isolated from forest soil. J Microbiol. 2008, 46:396-401.

8. Pellegrino FLPC, Vieira VV, Baio PVP, dos Santos RMR, dos Santos ALA, Santos NGdB, Meohas MMGL, Santos RT, de Souza TC, da Silva Dias RC, et al: *Acinetobacter soli* as a cause of bloodstream infection in a neonatal intensive care unit. J Clin Microbiol. 2011, 49:2283-2285.

9. Liu C, Li Y, Ye L, Zhao J, Piao C, Li Z, Li J, Xiang W, Wang X: *Actinocorallia lasiicapitis* sp. nov., an actinomycete isolated from the head of an ant (*Lasius fuliginosus* L.). Int J Syst Evol Microbiol. 2016, 66:2172-2177.

10. Itoh T, Kudo T, Oyaizu H, Seino A: Two New Species in the Genus *Actinomadura*: A. glomerata sp. nov., and A. longicatena sp. nov. Actinomycetologica. 1995, 9:164-177.

11. Casida Jr L: Interaction of *Agromyces ramosus* with other bacteria in soil. Appl Environ Microbiol. 1983, 46:881-888.

12. Kwong WK, Moran NA: *Apibacter adventoris* gen. nov., sp. nov., a member of the phylum Bacteroidetes isolated from honey bees. Int J Syst Evol Microbiol. 2016, 66:1323-1329.

13. Makk J, Homonnay ZG, Keki Z, Nemes-Barnas K, Marialigeti K, Schumann P, Toth EM: *Arenimonas subflava* sp. nov., isolated from a drinking water network, and emended description of the genus *Arenimonas*. Int J Syst Evol Microbiol. 2015, 65:1915-1921.

14. Sly LI, Cox TL, Beckenham TB: The phylogenetic relationships of *Caulobacter*, *Asticcacaulis* and *Brevundimonas* species and their taxonomic implications. Int J Syst Bacteriol. 1999, 49 Pt 2:483-488.

15. Thongaram T, Kosono S, Ohkuma M, Hongoh Y, Kitada M, Yoshinaka T, Trakulnaleamsai S, Noparatnaraporn N, Kudo T: Gut of Higher Termites as a Niche for Alkaliphiles as Shown by Culture-Based and Culture-Independent Studies. Microbes Environ. 2003, 18:152-159.

16. Borsodi A, Micsinai A, Rusznyák A, Vladár P, Kovacs G, Toth E, Marialigeti KJMe: Diversity of alkaliphilic and alkalitolerant bacteria cultivated from decomposing reed rhizomes in a Hungarian soda lake. Microb. Ecol. 2005, 50:9-18.

17. Ki JS, Zhang W, Qian PY: Discovery of marine Bacillus species by 16S rRNA and rpoB comparisons and their usefulness for species identification. Microbiol Methods. 2009, 77:48-57.

18. Suresh K, Prabagaran SR, Sengupta S, Shivaji S: Bacillus indicus sp. nov., an arsenic-resistant bacterium isolated from an aquifer in West Bengal, India. Int J Syst Evol Microbiol. 2004, 54:1369-1375.

19. Manzo N, D'Apuzzo E, Coutinho PM, Cutting SM, Henrissat B, Ricca E: Carbohydrate-active enzymes from pigmented Bacilli: a genomic approach to assess carbohydrate utilization and degradation. BMC Microbiol. 2011, 11:198.

20. Hong HA, Huang JM, Khaneja R, Hiep LV, Urdaci MC, Cutting SM: The safety of *Bacillus subtilis* and *Bacillus indicus* as food probiotics. Appl Microbiol. 2008, 105:510-520.

21. Venkateswaran K, Kempf M, Chen F, Satomi M, Nicholson W, Kern R: *Bacillus nealsonii* sp. nov., isolated from a spacecraft-assembly facility, whose spores are gamma-radiation resistant. Int J Syst Evol Microbiol. 2003, 53:165-172.

22. Phulpoto IA, Yu Z, Hu B, Wang Y, Ndayisenga F, Li J, Liang H, Qazi MA: Production and characterization of surfactin-like biosurfactant produced by novel strain *Bacillus nealsonii* S2MT and it's potential for oil contaminated soil remediation. Microb. Cell Factories. 2020, 19:145.

23. Farooqi A, Din G, Hayat R, Badshah M, Khan S, Shah AA: Characterization of *Bacillus nealsonii* strain KBH10 capable of reducing aqueous mercury in laboratory-scale reactor. Water Sci Technol. 2021, 83:2287-2295.

24. Fresco-Taboada A, Del Cerro C, Fernández-Lucas J, Arroyo M, Acebal C, García JL, de la Mata I: Genome of the Psychrophilic Bacterium *Bacillus psychrosaccharolyticus*, a Potential Source of 2'-Deoxyribosyltransferase for Industrial Nucleoside Synthesis. Genome Announc. 2013, 1.

25. Combet-Blanc Y, Ollivier B, Streicher C, Patel B, Dwivedi P, Pot B, Prensier G, Garcia J-L: *Bacillus thermoamylovorans* sp. nov., a moderately thermophilic and amylolytic bacterium. Int J Syst Evol Microbiol. 1995, 45:9-16.

26. Yohandini H, Julinar, Muharni: Isolation and Phylogenetic Analysis of Thermophile Community Within Tanjung Sakti Hot Spring, South Sumatera, Indonesia. HAYATI J Biosci. 2015, 22:143-148.

27. Flint S, Gonzaga ZJ, Good J, Palmer J: *Bacillus thermoamylovorans*–A new threat to the dairy industry–A review. Int Dairy J. 2017, 65:38-43.

28. Pham VHT, Jeong S, Chung S, Kim J: *Brevundimonas albigilva* sp. nov., isolated from forest soil. Int J Syst Evol Microbiol. 2016, 66:1144-1150.

29. Tsubouchi T, Shimane Y, Usui K, Shimamura S, Mori K, Hiraki T, Tame A, Uematsu K, Maruyama T, Hatada Y: *Brevundimonasabyssalis* sp. nov., a dimorphic prosthecate bacterium isolated from deep-subsea floor sediment. Int J Syst Evol Microbiol. 2013, 63:1987-1994.

30. Estrela AB, Abraham W-R: *Brevundimonas vancanneytii* sp. nov., isolated from blood of a patient with endocarditis. Int J Syst Evol Microbiol. 2010, 2129-2134.

31. Naqqash T, Imran A, Hameed S, Shahid M, Majeed A, Iqbal J, Hanif MK, Ejaz S, Malik KA: First report of diazotrophic *Brevundimonas* spp. as growth enhancer and root colonizer of potato. Sci Rep. 2020, 10:12893.

32. Rathi M, K NY: *Brevundimonas diminuta* MYS6 associated *Helianthus annuus* L. for enhanced copper phytoremediation. Chemosphere. 2021, 263:128195.

33. Ryan MP, Pembroke JT: *Brevundimonas* spp: Emerging global opportunistic pathogens. Virulence. 2018, 9:480-493.

34. Jiang L, Jeon D, Kim J, Lee CW, Peng Y, Seo J, Lee JH, Paik JH, Kim CY, Lee J: Pyomelanin-Producing *Brevundimonas vitisensis* sp. nov., Isolated From Grape (*Vitis vinifera* L.). Front Microbiol. 2021, 12; 73612.

35. Tegtmeier D, Riese C, Geissinger O, Radek R, Brune A: *Breznakia blatticola* gen. nov. sp. nov. and *Breznakia pachnodae* sp. nov., two fermenting bacteria isolated from insect guts, and emended description of the family Erysipelotrichaceae. Syst Appl Microbiol. 2016, 39:319-329.

36. Mannaa M, Park I, Seo YS: Genomic Features and Insights into the Taxonomy, Virulence, and Benevolence of Plant-Associated Burkholderia Species. Int J Mol Sci. 2018, 20.

37. Sadauskas M, Statkevičiūtė R, Vaitekūnas J, Meškys R: Bioconversion of Biologically Active Indole Derivatives with Indole-3-Acetic Acid-Degrading Enzymes from *Caballeronia glathei* DSM50014. Biomolecules 2020, 10:663.

38. Leisner JJ, Laursen BG, Prévost H, Drider D, Dalgaard P: Carnobacterium: positive and negative effects in the environment and in foods. FEMS Microbiol Rev. 2007, 31:592-613.

39. Lee SD, Kang SO, Hah YC: *Catellatospora koreensis* sp. nov., a novel actinomycete isolated from a gold-mine cave. Int J Syst Evol Microbiol. 2000, 50 Pt 3:1103-1111.

40. Chang SC, Wu MC, Chen WM, Tsai YH, Lee TM: *Chitinilyticum litopenaei* sp. nov., isolated from a freshwater shrimp pond, and emended description of the genus *Chitinilyticum*. Int J Syst Evol Microbiol. 2009, 59:2651-2655.

41. Wisplinghoff H: *Pseudomonas* spp., *Acinetobacter* spp. and miscellaneous Gram-negative bacilli. In Infectious diseases. Elsevier; 2017: 1579-1599. e1572

42. Mata-Cabana A P-NC, Olmedo M: Nutritional control of postembryonic development progression and arrest in Caenorhabditis elegans. In Advances in Genetics. Volume 107. Edited by Kumar D: Academic Press; 2021: 33-87

43. Chang YH, Han JI, Chun J, Lee KC, Rhee MS, Kim YB, Bae KS: *Comamonas koreensis* sp. nov., a non-motile species from wetland in Woopo, Korea. Int J Syst Evol Microbiol. 2002, 52:377-381.

44. Wu C-Y, Zhuang L, Zhou S-G, Li F-B, Li X-M: Fe(III)-enhanced anaerobic transformation of 2,4-dichlorophenoxyacetic acid by an iron-reducing bacterium *Comamonas koreensis* CY01. FEMS Microbiol Ecol. 2009, 71:106-113.

45. Chou JH, Sheu SY, Lin KY, Chen WM, Arun AB, Young CC: *Comamonas odontotermitis* sp. nov., isolated from the gut of the termite *Odontotermes formosanus*. Int J Syst Evol Microbiol. 2007, 57:887-891.

46. Firdous S, Iqbal S, Anwar S: Optimization and Modeling of Glyphosate Biodegradation by a Novel *Comamonas odontotermitis* P2 Through Response Surface Methodology. Pedosphere. 2017, 30.

47. Subhash Y, Bang JJ, You TH, Lee SS: Description of *Comamonas sediminis* sp. nov., isolated from lagoon sediments. Int J Syst Evol Microbiol. 2016, 66:2735-2739.

48. Xing Z, Hu T, Xiang Y, Qi P, Huang X: Degradation Mechanism of 4-Chlorobiphenyl by Consortium of *Pseudomonas* sp. Strain CB-3 and *Comamonas* sp. Strain CD-2. Curr Microbiol. 2020, 77:15-23.

49. Zhu Y, McBride MJ: The unusual cellulose utilization system of the aerobic soil bacterium Cytophaga hutchinsonii. Appl Microbiol Biotechnol. 2017, 101:7113-7127.

50. Xie G, Bruce DC, Challacombe JF, Chertkov O, Detter JC, Gilna P, Han CS, Lucas S, Misra M, Myers GL, et al: Genome sequence of the cellulolytic gliding bacterium *Cytophaga hutchinsonii*. Appl Environ Microbiol. 2007, 73:3536-3546.

51. Shashidhar R, Bandekar JR: *Deinococcus piscis* sp. nov., a radiation-resistant bacterium isolated from a marine fish. Int J Syst Evol Microbiol. 2009, 59:2714-2717.

52. Sharma D, Patel A, Soni P, Sharma P, Gupta B: *Empedobacter brevis* Meningitis in a Neonate: A Very Rare Case of Neonatal Meningitis and Literature Review. Case Rep Pediatr. 2016, 2016:7609602.

53. Zhai H, Zhao Y, Gao X, Wu MJBDS: Efficacy of *Empedobacter brevis* in controlling rice stem borer. Biol Disast Sci. 2013, 36:291-294.

54. Dolka B, Chrobak-Chmiel D, Czopowicz M, Szeleszczuk P: Characterization of pathogenic *Enterococcus cecorum* from different poultry groups: Broiler chickens, layers, turkeys, and waterfowl. PLoS One. 2017, 12:e0185199.

55. Jung A, Metzner M, Ryll M: Comparison of pathogenic and non-pathogenic *Enterococcus cecorum* strains from different animal species. BMC Microbiol. 2017, 17:33.

56. Vela AI, Fernandez A, Sánchez-Porro C, Sierra E, Mendez M, Arbelo M, Ventosa A, Domínguez L, Fernández-Garayzábal JF: *Flavobacterium ceti* sp. nov., isolated from beaked whales (Ziphius cavirostris). Int J Syst Evol Microbiol. 2007, 57:2604-2608.

57. Park S-K, Ryoo N: A Case of *Flavobacterium ceti* Meningitis. Ann Lab Med. 2016, 36:614.

58. Saha P, Chakrabarti T: *Flavobacterium indicum* sp. nov., isolated from warm spring water in Assam, India. Int J Syst Evol Microbiol. 2006, 56:2617-2621.

59. Barbier P, Houel A, Loux V, Poulain J, Bernardet JF, Touchon M, Duchaud E: Complete genome sequence of *Flavobacterium indicum* GPSTA100-9T, isolated from warm spring water. Bacteriol. 2012, 194:3024-3025.

60. Lata P, Lal D, Lal R: *Flavobacterium ummariense* sp. nov., isolated from hexachlorocyclohexane-contaminated soil, and emended description of *Flavobacterium ceti* Vela et al. 2007. Int J Syst Evol Microbiol. 2012, 62:2674-2679.

61. Chang X, Zheng J, Jiang F, Liu P, Kan W, Qu Z, Fang C, Peng F: *Hymenobacter arcticus* sp. nov., isolated from glacial till. Int J Syst Evol Microbiol. 2014, 64:2113-2118.

62. Martin RM, Bachman MA: Colonization, Infection, and the Accessory Genome of *Klebsiella pneumoniae*. Front Cell Infect Microbiol. 2018, 8.4.

63. Ji SH, Gururani MA, Chun S-C: Isolation and characterization of plant growth promoting endophytic diazotrophic bacteria from Korean rice cultivars. Microbiol Res. 2014, 169:83-98.

64. Lombogia CA, Tulung M, Posangi J, Tallei TE: Bacterial Composition, Community Structure, and Diversity in *Apis nigrocincta* Gut. Int J Microbiol. 2020, 2020:6906921.

65. Smith CC, Srygley RB, Healy F, Swaminath K, Mueller UG: Spatial Structure of the Mormon Cricket Gut Microbiome and its Predicted Contribution to Nutrition and Immune Function. Front Microbiol. 2017, 8, 801.

66. Gupta AK, Nayduch D, Verma P, Shah B, Ghate HV, Patole MS, Shouche YS: Phylogenetic characterization of bacteria in the gut of house flies (*Musca domestica* L.). FEMS Microbiol Ecol. 2012, 79:581-593.

67. Lundgren JG, Lehman RM: Bacterial Gut Symbionts Contribute to Seed Digestion in an Omnivorous Beetle. PLoS One. 2010, 5:e10831.

68. Wang FQ, Wang ET, Liu J, Chen Q, Sui XH, Chen WF, Chen WX: Mesorhizobium albiziae sp. nov., a novel bacterium that nodulates *Albizia kalkora* in a subtropical region of China. Int J Syst Evol Microbiol. 2007, 57:1192-1199.

69. Laranjo M, Oliveira S: Tolerance of *Mesorhizobium* type strains to different environmental stresses. Antonie Van Leeuwenhoek. 2011, 99:651-662.

70. Dastager SG, Lee J-C, Ju Y-J, Park D-J, Kim C-J: *Nocardioides halotolerans* sp. nov., isolated from soil on Bigeum Island, Korea. Syst Appl Microbiol. 2008, 31:24-29.

71. Jensen H: Studies on saprophytic *Mycobacterium* and *Corynebacterium*. Proc. Linn. Soc. N. S. W. 1934: 19-61.

72. Shtratnikova VY, Sсhelkunov MI, Fokina VV, Bragin EY, Shutov AA, Donova MV: Different genome-wide transcriptome responses of *Nocardioides simplex* VKM Ac-2033D to phytosterol and cortisone 21-acetate. BMC Biotechnol. 2021, 21:7.

73. Anderson-Glenna M, Bakkestuen V, Clipson N: Anderson-Glenna MJ, Bakkestuen V, Clipson NJWSpatial and temporal variability in epilithic biofilm bacterial communities along an upland river gradient. FEMS Microbiol Ecol. 2008, 64: 407-418.

74. Bousbia S, Papazian L, Saux P, Forel J-M, Auffray J-P, Martin C, Raoult D, La Scola B: Serologic Prevalence of Amoeba-Associated Microorganisms in Intensive Care Unit Pneumonia Patients. PLoS One. 2013, 8:e58111.

75. Liu X, Hou W, Dong H, Wang S, Jiang H, Wu G, Yang J, Li G. Distribution and diversity of *Cyanobacteria* and eukaryotic algae in Qinghai–Tibetan lakes. Geomicrobiol J. 2016; 25;33:860-869.

76. Amagata T: Natural Products Structural Diversity-II Secondary Metabolites: Sources, Structures and Chemical Biology. Comp Nat Pro II. 2010, 2:581-621.

77. Kellner RLL, Dettner K: Allocation of pederin during lifetime of *Paederus* rove beetles (Coleoptera: Staphylinidae): Evidence for polymorphism of hemolymph toxin. J Chem Ecol. 1995, 21:1719-1733.

78. Shin SK, Kim E, Yi H: *Paenibacillus crassostreae* sp. nov., isolated from the Pacific oyster *Crassostrea gigas*. Int J Syst Evol Microbiol. 2018, 68:58-63.

79. Park DS, Jeong WJ, Lee KH, Oh HW, Kim BC, Bae KS, Park HY: *Paenibacillus pectinilyticus* sp. nov., isolated from the gut of *Diestrammena apicalis*. Int J Syst Evol Microbiol. 2009, 59:1342-1347.

80. Davidov Y, Jurkevitch E: Diversity and evolution of Bdellovibrio-and-like organisms (BALOs), reclassification of *Bacteriovorax starrii* as *Peredibacter starrii* gen. nov., comb. nov., and description of the *Bacteriovorax*–*Peredibacter* clade as Bacteriovoracaceae fam. nov. FEMS Microbiol Ecol. 2004, 54:1439-1452.

81. Hokmolahi F, Riahi H, Soltani N, Shariatmadari Z, Hakimi Meibodi MH: A taxonomic study on non-heterocystous filamentous Cyanoprokaryotes from soil of Yazd province, Iran. Iran J Botan. 2017, 23:60-71.

82. Hahn MW, Lang E, Brandt U, Lünsdorf H, Wu QL, Stackebrandt E: *Polynucleobacter cosmopolitanus* sp. nov., free-living planktonic bacteria inhabiting freshwater lakes and rivers. Int J Syst Evol Microbiol. 2010, 60:166-173.

83. Könönen E, Gursoy UK: Oral Prevotella species and their connection to events of clinical relevance in gastrointestinal and respiratory tracts. Front Microbiol 2021, 12.

84. Prasoodanan P. K V, Sharma AK, Mahajan S, Dhakan DB, Maji A, Scaria J, Sharma VK: Western and non-western gut microbiomes reveal new roles of *Prevotella* in carbohydrate metabolism and mouth–gut axis. npj Biofilms and Microbiomes. 2021, 7:77.

85. Cuesta G, Soler A, Alonso JL, Ruvira MA, Lucena T, Arahal DR, Goodfellow M: *Pseudonocardia hispaniensis* sp. nov., a novel actinomycete isolated from industrial wastewater activated sludge. Antonie Van Leeuwenhoek. 2013, 103:135-142.

86. Riahi HS, Heidarieh P, Fatahi-Bafghi M: Genus Pseudonocardia: What we know about its biological properties, abilities and current application in biotechnology. J Appl Microbiol. 2022, 132:890-906.

87. Aremu BR, Babalola OO: Classification and taxonomy of vegetable macergens. Front Microbiol. 2015, 6:1361.

88. Joanna María O-A, José Miguel S-C, Fabiola G-A, Elizabeth G-D, Araceli R-C, Patricia A-P, Claudia W-A, Maribel G-V, Gloria L-Á, Adda Jeanette G-C: Fatal Psychrobacter sp. infection in a pediatric patient with meningitis identified by metagenomic next-generation sequencing in cerebrospinal fluid. Arch Microbiol. 2016, 198:129-135.

89. Maszenan AM, Tay JH, Schumann P, Jiang H-L, Tay ST-l: *Quadrisphaera granulorum* gen. nov., sp. nov., a Gram-positive polyphosphate-accumulating coccus in tetrads or aggregates isolated from aerobic granules. Int J Syst Evol Microbiol. 2005, 55 Pt 5:1771-1777.

90. Rowbotham TJ, Cross T: *Rhodococcus coprophilus* sp. nov.: An Aerobic *Nocardioform Actinomycete* belonging to the ‘*Rhodochrous*’ complex. Microbiol. 1977, 100:123-138.

91. Mara DD, Oragui JI: Occurrence of *Rhodococcus coprophilus* and associated Actinomycetes in feces, sewage, and freshwater. Appl Environ Microbiol. 1981, 42:1037-1042.

92. Vereecke D, Zhang Y, Francis IM, Lambert PQ, Venneman J, Stamler RA, Kilcrease J, Randall JJ: Functional genomics insights into the pathogenicity, habitat fitness, and mechanisms modifying plant development of *Rhodococcus* sp. PBTS1 and PBTS2. Front Microbiol. 2020, 11.

93. Ahmad M, Roberts JN, Hardiman EM, Singh R, Eltis LD, Bugg TD: Identification of DypB from *Rhodococcus jostii* RHA1 as a lignin peroxidase. Biochem. 2011, 50:5096-5107.

94. Kim DU, Lee H, Kim SG, Ka JO: *Roseomonas terricola* sp. nov., isolated from agricultural soil. Int J Syst Evol Microbiol. 2017, 67:4836-4841.

95. Das A, Panda A, Singh D, Chandrababunaidu MM, Mishra GP, Bhan S, Adhikary SP, Tripathy S: Deciphering the genome sequences of the hydrophobic *Cyanobacterium scytonema* tolypothrichoides VB-61278. Genome Announc. 2015, 3.

96. Harmon-Smith M, Celia L, Chertkov O, Lapidus A, Copeland A, Glavina Del Rio T, Nolan M, Lucas S, Tice H, Cheng JF, et al: Complete genome sequence of *Sebaldella termitidis* type strain (NCTC 11300). Stand Genomic Sci. 2010, 2:220-227.

97. Weon HY, Kim BY, Lee CM, Hong SB, Jeon YA, Koo BS, Kwon SW: *Solitalea koreensis* gen. nov., sp. nov. and the reclassification of [*Flexibacter*] *canadensis* as *Solitalea canadensis* comb. nov. Int J Syst Evol Microbiol. 2009, 59:1969-1975.

98. Cortes-Tolalpa L, Wang Y, Salles JF, van Elsas JD: Comparative genome analysis of the lignocellulose degrading bacteria *Citrobacter freundii* so4 and *Sphingobacterium multivorum* w15. Front Microbiol. 2020, 11:248.

99. Yabuuchi E, Kaneko T, Yano I, Moss CW, Miyoshi N: *Sphingobacterium* gen. nov., *Sphingobacterium spiritivorum* comb. nov., *Sphingobacterium multivorum* comb. nov., *Sphingobacterium mizutae* sp. nov., and *Flavobacterium indologenes* sp. nov.: glucose-nonfermenting gram-negative rods in CDC groups IIK-2 and IIb. Int J Syst Evol Microbiol. 1983, 33:580-598.

100. Sahar N, Shahid M, Ali A: A case of *Sphingobacterium spiritivorum* bacteremia and literature review. Infect Dis Clin Pract. 2020, 28:7-9.

101. Garcia-Lopez M-L, Santos, J.-A., Otero, A: *Flavobacterium*, in encyclopedia of food microbiology. In Volume II. San Diego, California: Academic Press; 2000

102. Yabe S, Aiba Y, Sakai Y, Hazaka M, Kawahara K, Yokota A: Sphingobacterium thermophilum sp. nov., of the phylum Bacteroidetes, isolated from compost. Int J Syst Evol Microbiol. 2013, 63:1584-1588.

103. Busse HJ, Hauser E, Kämpfer P: Description of two novel species, *Sphingomonas abaci* sp. nov. and *Sphingomonas panni* sp. nov. Int J Syst Evol Microbiol. 2005, 55:2565-2569.

104. Kim H-B, Park M-J, Yang H-C, An D-S, Jin H-Z, Yang D-C: *Burkholderia ginsengisoli* sp. nov., a β-glucosidase-producing bacterium isolated from soil of a ginseng field. Int J Syst Evol Microbiol. 2006, 56:2529-2533.

105. Finkmann W, Altendorf K, Stackebrandt E, Lipski A: Characterization of N2O-producing Xanthomonas-like isolates from biofilters as *Stenotrophomonas nitritireducens* sp. nov., *Luteimonas mephitis* gen. nov., sp. nov. and *Pseudoxanthomonas broegbernensis* gen. nov., sp. nov. Int J Syst Evol Microbiol. 2000, 50:273-282.

106. Wolf A, Fritze A, Hagemann M, Berg G: *Stenotrophomonas rhizophila* sp. nov., a novel plant-associated bacterium with antifungal properties. Int J Syst Evol Microbiol. 2002, 52:1937-1944.

107. Kämpfer P, Lindh JM, Terenius O, Haghdoost S, Falsen E, Busse H-J, Faye I: *Thorsellia anophelis* gen. nov., sp. nov., a new member of the Gammaproteobacteria. Int J Syst Evol Microbiol. 2006, 56:335-338.

108. Briones AM, Shililu J, Githure J, Novak R, Raskin L: *Thorsellia anophelis* is the dominant bacterium in a Kenyan population of adult *Anopheles gambiae* mosquitoes. The ISME J. 2008, 2:74-82.

109. Kämpfer P, Glaeser SP, Nilsson LK, Eberhard T, Håkansson S, Guy L, Roos S, Busse H-J, Terenius O: Proposal of *Thorsellia kenyensis* sp. nov. and *Thorsellia kandunguensis* sp. nov., isolated from larvae of *Anopheles arabiensis*, as members of the family Thorselliaceae fam. nov. Int J Syst Evol Microbiol. 2015, 65:444-451.
